# A novel patient-derived 3D model recapitulates mantle cell lymphoma lymph node signaling, immune profile and in vivo ibrutinib responses

**DOI:** 10.1038/s41375-023-01885-1

**Published:** 2023-04-08

**Authors:** Ferran Araujo-Ayala, Cèlia Dobaño-López, Juan García Valero, Ferran Nadeu, Fabien Gava, Carla Faria, Marine Norlund, Renaud Morin, Pascale Bernes-Lasserre, Neus Serrat, Heribert Playa-Albinyana, Rubén Giménez, Elías Campo, Jean-Michel Lagarde, Armando López-Guillermo, Eva Gine, Dolors Colomer, Christine Bezombes, Patricia Pérez-Galán

**Affiliations:** 1 Fundació de Recerca Clínic Barcelona (FCRB)-IDIBAPS, Barcelona, Spain; 2 Centro de Investigación Biomédica en Red-Oncología (CIBERONC), Madrid, Spain; 3 Centre de Recherches en Cancérologie de Toulouse (CRCT), INSERM UMR1037, Toulouse, France; 4 Université de Toulouse, Inserm, CNRS, Université Toulouse IIIPaul Sabatier, Centre de Recherches en Cancérologie de Toulouse, Toulouse, France; 5 IUCT-Oncopole, Toulouse, France; 6 Laboratoire d’Excellence ‘TOUCAN-2’, Toulouse, France; 7 Institut Carnot Lymphome CALYM, Pierre-Bénite, France; 8 IMACTIV-3D, Toulouse, France; 9 Hospital Clínic, Barcelona, Spain; 10 University of Barcelona, Medical School, Barcelona, Spain

**Keywords:** Cancer models, Cancer microenvironment, B-cell lymphoma

## Abstract

Mantle cell lymphoma (MCL), a rare and aggressive B-cell non-Hodgkin lymphoma, mainly develops in the lymph node (LN) and creates a protective and immunosuppressive niche that facilitates tumor survival, proliferation and chemoresistance. To capture disease heterogeneity and tumor microenvironment (TME) cues, we have developed the first patient-derived MCL spheroids (MCL-PDLS) that recapitulate tumor oncogenic pathways and immune microenvironment in a multiplexed system that allows easy drug screening, including immunotherapies. MCL spheroids, integrated by tumor B cells, monocytes and autologous T-cells self-organize in disc-shaped structures, where B and T-cells maintain viability and proliferate, and monocytes differentiate into M2-like macrophages. RNA-seq analysis demonstrated that tumor cells recapitulate hallmarks of MCL-LN (proliferation, NF-kB and BCR), with T cells exhibiting an exhaustion profile (PD1, TIM-3 and TIGIT). MCL-PDLS reproduces in vivo responses to ibrutinib and demonstrates that combination of ibrutinib with nivolumab (anti-PD1) may be effective in ibrutinib-resistant cases by engaging an immune response with increased interferon gamma and granzyme B release. In conclusion, MCL-PDLS recapitulates specific MCL-LN features and in vivo responses to ibrutinib, representing a robust tool to study MCL interaction with the immune TME and to perform drug screening in a patient-derived system, advancing toward personalized therapeutic approaches.

## Introduction

Mantle cell lymphoma (MCL) is a rare and aggressive B-cell non-Hodgkin lymphoma characterized by CCND1 deregulation caused by the t(11;14)(q13;q32) translocation as the first oncogenic hit. Moreover, MCL is characterized by genomic instability and high number of secondary genetic aberrations that are necessary to engage lymphomagenesis. MCL is an heterogeneous disease “per se” and World Health Organization recognizes 2 molecular subtypes that differ in their clinical and biological features: the most common and aggressive conventional MCL (cMCL, SOX11+ and unmutated immunoglobulin heavy chain (IGHV), naïve-like B-cell) and the indolent leukemic non-nodal MCL (nnMCL; SOX11− and mutated IGHV, memory-like B-cell) [1–3]. Besides, both forms differ in the underlying genomic and epigenomic abnormalities [4].

In the last decade, next generation sequencing studies have deciphered the MCL mutational landscape identifying recurrent mutations (*TP53, ATM, NOTCH1/2, CCND1, HNRNPH1, KMT2D, ARID1A*, *SMARCA4)* [5, 6] that contribute to MCL pathogenesis and resistance to chemoimmunotherapy or targeted therapies [7]. These genomic alterations often impact molecular pathways that are involved in DNA damage response, cell proliferation and cell survival [8, 9]. In addition to these genomic abnormalities it is fundamental to consider MCL-tumor microenvironment (TME) crosstalk within the lymph node (LN) [10, 11]. This dialog, together with genomic alterations, leads to the activation of MCL hallmarks pathways of cell proliferation, DNA repair, apoptosis inhibition, NF-kB and BCR signaling [12], with a different representation among patients. In this regard, a study combining genomic and transcriptomic profiling has revealed distinct patients subsets, grouped by genomic alterations and activated pathways [6], associated with differential outcomes, thus reflecting the cooperation between genome aberrations and TME on disease development and outcomes.

In the LN ecosystem, the interaction between MCL tumor cells and T cells through CD40L and IL-4 is fundamental to promote tumor proliferation and viability [13]. Likewise, stromal cells as follicular dendritic cells (FDC) [14, 15], through integrin receptors and secreted factors as CXCL12/13 or BAFF [16–18] maintain MCL viability. In addition, macrophages play a fundamental role in this scenario, as their number is associated with poor prognosis [19], support MCL cell growth in vitro [20] as well as in vivo [21], and may induce immune exhaustion through PD-L1 expression [22].

In view of the heterogeneity of this rare disease and the critical contribution of TME, powerful preclinical systems using patient-derived material and recapitulating microenvironment cues are mandatory. Several attempts have been described in order to maintain lymphoma patient-derived cultures and induce their proliferation in 2D co-culture systems [17, 23–25]. In the last years there has been an evolution toward patient-derived 3D cultures and organoids (PDO) in many cancer types [26]. However, those systems are scarce in lymphoma [27, 28] and not previously generated in MCL. In the era of personalized medicine and with the rapid evolution of immunotherapies, there is an urgent need to establish these systems that recapitulate disease activated pathway and immune profile, and are able to induce T-cell mediated responses. In this work, we aimed to develop a novel 3D spheroid-based model to culture MCL primary cells together with autologous T cells and healthy donor monocytes, recreating the immune TME. This MCL Patient Derived Lymphoma Spheroid (MCL-PDLS) reproduces a specific MCL-LN signature [12] making it a suitable tool to study MCL biology, and to test both conventional and immunotherapeutic drugs together with identification of biomarkers of response and relapse.

## Methods

### PDLS generation

Peripheral blood mononuclear cells (PBMCs) isolated from MCL (*n* = 19) samples were thawed in sterile conditions, resuspended in enriched medium [29] and counted using Neubauer chamber system with trypan blue to assess cell viability. In order to assess proliferation, cells were labeled with 0.5 μM carboxyfluorescein succinimidyl ester (CFSE) cell tracker (Thermo Fisher Scientific, Waltham, MA, USA) following manufacturer´s instructions.

**Fig. 1:**
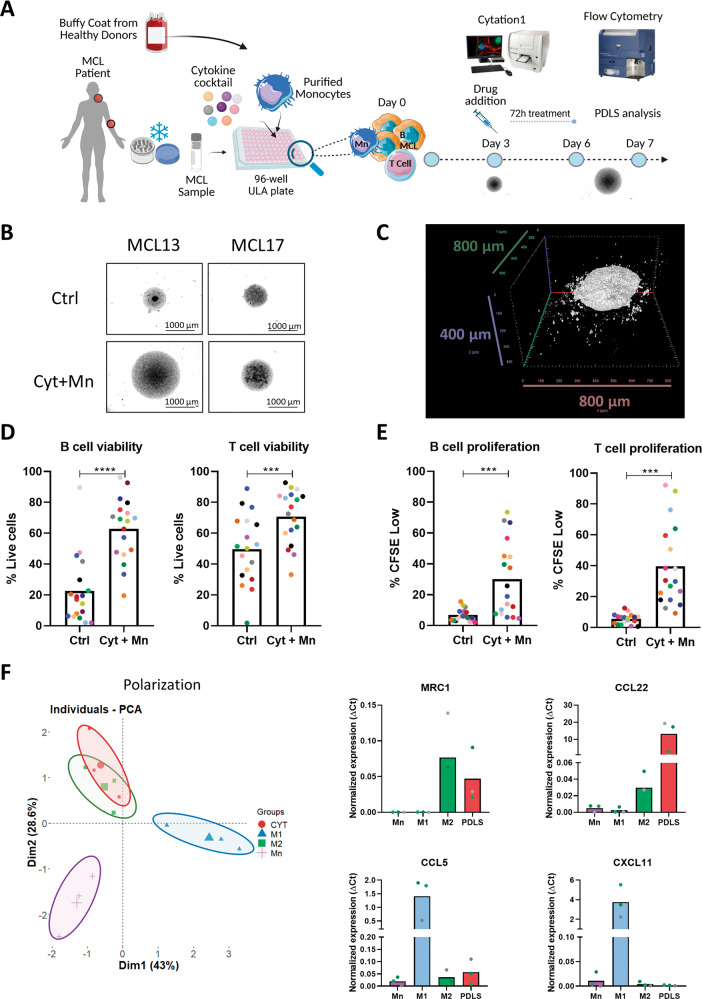
MCL-PDLS as a novel 3D model to culture MCL samples ex vivo. **A** Representative scheme showing the workflow for MCL-PDLS generation. Created with BioRender.com. **B** Brightfield images (magnification ×40) captured in the Cytation 1 of PDLS generated with cytokines (Cyt) and monocytes (Mn) stimuli compared to non-stimulated PDLS control (Ctrl) after 7 days of culture. **C** 3D reconstruction of a representative PDLS (MCL 1) from an image obtained by SPIM microscopy. **D** Cell viability in tumor B cells and autologous T cells from PDLS determined by percentage of negative LIVE/DEAD fixable Aqua staining (*n* = 18) after 7 days of culture. **E** Cell proliferation in B cells and T cells, calculated as percentage of CFSE low cells, after 7 days of culture (*n* = 18). **F** PCA analysis using normalized expression values of six genes related to macrophage polarization obtained by RT-qPCR in macrophages isolated from MCL-PDLS and 2D-differentiated macrophages polarized to M1 or M2 phenotype as references. Undifferentiated monocytes were used as a control. *MRC1* and *CCL22* are used as M2 markers while *CCL5* and *CXCL11* are used as M1 markers.

The workflow for MCL-PDLS generation is detailed in Fig. 1A. CFSE-labeled MCL samples were mixed with monocytes at a 4:1 ratio (MCL:monocytes), seeding 5 × 10^4^ MCL cells/well and 1.25 × 10^4^ monocytes/well in a final volume of 200 μl/well in Nunclon^TM^ Sphera^TM^ 96-wells Ultra-Low Attachment (ULA) microplates (Thermo Fisher Scientific) in enriched medium supplemented with the following cytokines: 50 ng/ml CD40L-HA tagged (R&D Systems, Minneapolis, MN, USA), 1 μg/ml anti-HA-Tag antibody (Merck, Darmstadt, Germany), 10 ng/ml IL-4 (Peprotech, Cranbury, NJ, USA) and 50 ng/ml B-cell activating factor (BAFF) (Miltenyi Biotec), referred hereafter as “PDLS medium” and maintained at 37 °C 5% CO_2_ up to 7 days.

### Drug assays

PDLS generated as indicated above were cultured in 150 μl of PDLS medium, drugs were added at day 3 (50 μl/well in PDLS medium). Ibrutinib (Selleck Chemicals LLC, Houston, TX, USA) was added to a final concentration of 500 nM and nivolumab (Selleck Chemicals LLC) at 10 µg/ml. Six PDLS replicates were assessed per each condition. After 3 days of treatment, MCL-PDLS were mechanically disaggregated and analyzed by flow cytometry (BD LSRFortessa SORP-HTS, BD Biosciences, Franklin Lakes, NY, USA) to assess cell viability (LIVE/DEAD Fixable Aqua) and cell population distribution (CD20, CD3, CD4, CD8). To determine cell number of viable cells, disaggregated PDLS were analyzed using a High Throughput Sampler (HTS) integrated in the flow cytometry reading a fix volume.

Detailed description of additional methods is included in the Supplementary Material. These materials include: patient samples, monocyte-macrophage differentiation and polarization analysis, PDLS immune profile and activation, RNA-seq, metadata comparative analysis and a table of antibodies used to characterize the populations by flow cytometry (Table S1).

## Results

### Patient-derived MCL spheroids: cellular composition, distribution and 3D structure

In secondary lymphoid organs (SLO) as the LN, lymphoma B cells are in close contact with cells of immune origin, including CD40L-expressing T cells, and macrophages together with endothelial and stromal cells [30]. Thus, in order to generate a system that recapitulates microenvironment cues in SLO, we cultured MCL samples (Table 1), most of them from PB, in an optimized medium (PDLS medium) containing CD40L, IL-4 and BAFF, which are known to be fundamental to mimic interactions with T cells and stromal cells [16, 17]. Macrophages are often not recovered from biopsies and the number of monocytes in Peripheral Blood (PB) is highly variable and does not correlate with macrophage infiltration of SLO in MCL [31]. For this reason, monocytes from healthy donors were also included to account for the myeloid compartment, fundamental in this pathology [20, 21]. This multicellular suspension was seeded in ULA plates, as shown in Fig. 1A, to facilitate cell aggregation and growth (Fig. 1B). Nineteen MCL-PB samples were used to successfully generate PDLS. Spheroid formation occurs in the first 24 h after seeding (Supplementary Video 1). SPIM microscopy demonstrated that these structures self-organize in a real spheroid, with a mean volume of 0.16 mm^3^ (MCL1) (Fig. 1C and Supplementary Video 2). As shown in Fig. S1A, B, after 7 days of culture the viability of lymphoma B cells (mean 22.5) and accompanying T cells (mean 49.7), was significantly increased by both the cytokine cocktail (mean B cells 62.5; mean T cells 57.8) and the monocytes (mean B cells 55.45; mean T cells 73.92), separately. However, B-cell proliferation was only engaged by cytokines (mean 32.95). In the case of T cells, both cytokines (mean 21.47), and monocytes (mean 23.64) induced proliferation (Fig. S1A, B), albeit with a great variability and similarly to previous studies in 2D MCL stimulated with other cocktails [17]. This proliferation was also reflected in the spheroid diameter (Fig. S1C). Interestingly, this cytokine cocktail not only induced B-cell proliferation, but also activation, as revealed by flow cytometry changes in size (FSC) and complexity (SSC) and by the upregulation of CD69 and CD86, as seen in SLO (Fig. S1D). Thus, we chose to combine both cytokine cocktail and monocytes with the lymphoma B cells and T cells, which similarly increased viability in B and T cells (mean 62.88 and 70.9, respectively) and proliferation in B cells (mean 30.17), while improved the T-cell proliferation induced by cytokines alone (mean 39.57) (Fig. 1D, E). After 7 days, MCL PDLS were mostly composed of B cells (mean 82%) and similar proportion of T cells (mean 9,8%) and monocytes (mean 8,2%) (Fig. S1E).

**Table 1:** MCL patient characteristics.

Study label	Sex/Age^a^	Sample type^b^	% IgVH homology^c^	Stage^d^	MCL variant^e^	Disease status^f^	MIPI^g^	nnMCL/cMCL^h^	*TP53* altered^i^	Color
MCL1	M/63	PB	96.18^k^	IV	NA	R	Medium	nnMCL	Y	
MCL2	M/76	PB	97.92^j^	IV	C	D	High	cMCL	Y	
MCL4	M/NA	PB	98.11^j^	IV	NA	D	High	cMCL	NA	
MCL5	M/73	PB	99.6^j^	IV	NA	D	NA	cMCL	NA	
MCL6	M/43	PB	99.65^j^	IV	B	D	High	cMCL	NA	
MCL8	M/NA	PB	NA	NA	B	NA	NA	NA	NA	
MCL10	F/73	PB	100^j^	IV	C	R	High	cMCL	NA	
MCL11	M/71	PB	100^j^	IV	C	D	High	cMCL	N	
MCL12	M/64	PB	100^k^	IV	C	R	High	cMCL	N	
MCL13	M/70	PB	98.60^k^	IV	C	R	High	cMCL	N	
MCL14	M/75	PB	95.14^k^	IV	NA	R	High	nnMCL	Y	
MCL16	M/80	PB	97.22^k^	IV	NA	D	High	nnMCL	Y	
MCL17	F/78	PB	95.83^k^	NA	NA	R	NA	nnMCL	N	
MCL18	M/65	PB	91.58^k^	IV	NA	Pt	Medium	nnMCL	N	
MCL19	F/60	BM	97.32^j^	NA	NA	Pt	NA	nnMCL	NA	
MCL20	M/52	PB	NA	I	NA	R	Low	nnMCL	NA	
MCL21	F/59	PB	96.18^j^	IV	C	R	Low	cMCL	N	
MCL22	M/60	BM	93.47^k^	IV	NA	R	Medium	nnMCL	Y	
MCL24	F/59	LN	NA	IV	C	R	Low	cMCL	NA	

^a^F: female, M: male.

^b^PB: peripheral blood, LN: lymph node, BM: bone marrow.

^c^% of homology with the germline, assessed by ^j^Sanger sequencing or ^k^IgCaller [59].

^d^Ann Arbor stage.

^e^Evaluated by two independent pathologists. C: conventional, B: blastoid; NA cases did not have tissue available.

^f^Samples were obtained at D: diagnosis, R: relapse, Pt: pretreatment, NA: not available.

^g^MIPI: Mantle cell Lymphoma International Prognostic Index (High: 6–11; Medium: 4–5; Low: 0–3).

^h^nnMCL: non-nodal MCL; cMCL: conventional MCL.

^i^Mutated and/or deleted at the time of sampling. Y: yes, N: no, NA: not available.

It has been previously recognized that MCL cells induce differentiation of monocytes to M2 macrophages [12, 21]. Similarly, we observed that monocytes progressively differentiate into macrophages as shown by the increase in size and complexity (FSC/SSC) (Fig. S1F). These macrophages displayed high expression of M2-like markers as the mannose receptor *CD206* (*MRC1*) and *CCL22* (Fig. 1F), while M1-like markers were underrepresented (Fig. 1F). Moreover, the macrophages from the MCL-PDLS clearly clustered with M2-like macrophages in the PCA analysis generated with the expression of monocytes (*PMAIP1* and *RGS2*), M1 (*CCL5* and *CXCL1*) and M2 markers (*CCL22* and *MRC1*). These genes were selected from studies analyzing their differential expression [32, 33].

In summary, we have established for the first time a patient-derived 3D system integrating fundamental cellular and signaling component of MCL-TME with viable and proliferative B and T cells.

### Patient-derived MCL spheroids recapitulate MCL-LN signaling pathways

**Fig. 2:**
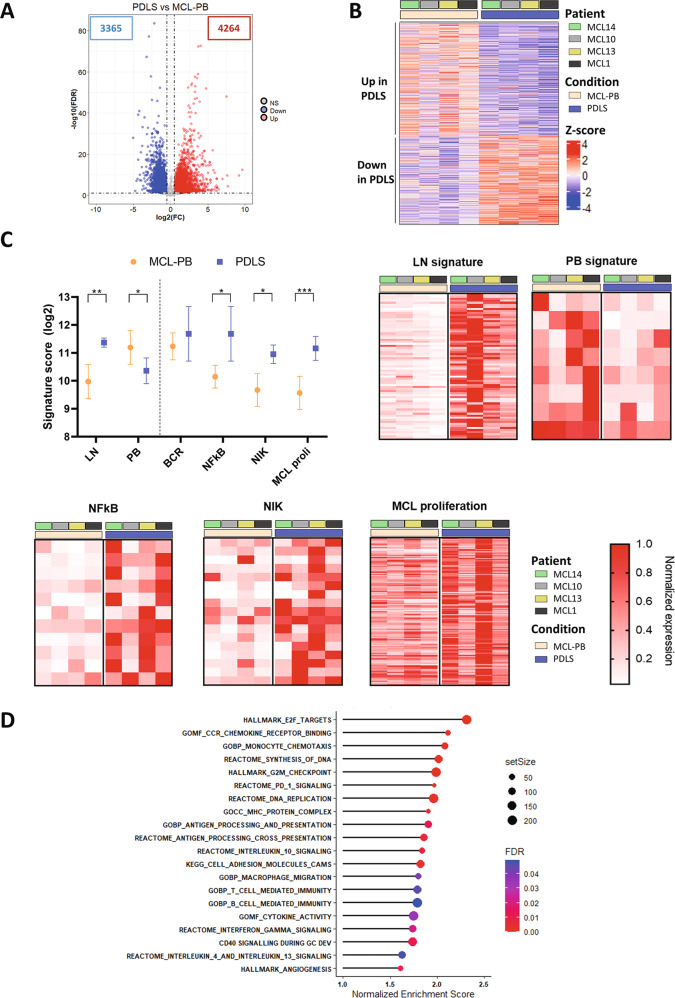
MCL-PDLS transcriptome recapitulates lymph node hallmarks. **A** Volcano plot representing the differentially expressed genes (DEG) comparing PDLS after 7 days of culture with the original MCL peripheral blood (MCL-PB) sample. DEG were obtained by a paired (*n* = 4) DESeq2 analysis (FDR < 0.1 and absolute log_2_FC > 0.5). **B** Heatmap of DEG for the individual patients (*n* = 4). **C** Signature score of MCL hallmark pathways as described by Saba and Rosenwald [12, 34]. Values were calculated as the geometric mean of the normalized counts for the genes involved in each pathway. For each significantly upregulated gene set, the leading genes are represented in a heatmap. **D** Bubble plot representing the most significant and representative GSEA pathways upregulated in MCL-PDLS compared to MCL-PB.

We next sought to determine if these MCL-PDLS engage a transcriptional program close to that of LN-resident MCL cells. Thus, we performed RNA-seq of purified B cells from unstimulated samples (MCL-PB) and compared with B cells isolated from the generated PDLS after 7 days of culture (*n* = 4). Differential expression analysis of paired samples indicated that 4262 genes were upregulated and 3365 downregulated in the PDLS (Fig. 2A, B), highlighting a significant transcriptome modulation. We next proceeded to validate if MCL-PDLS recapitulate MCL-LN signaling pathways. A recent study from Saba [12] and cols established MCL-PB and MCL-LN compartment-specific signatures. Using these signatures, as well as BCR, NF-kB and NIK pathway and proliferation signatures [12, 34], we demonstrated that the expression levels of the genes involved in those signatures (signature score) were significantly upregulated in the MCL-PDLS compared to original MCL-PB, while PB signature was downregulated. The leading-edge genes of these signatures are presented in a heatmap (Fig. 2C) and gene names are included in Table S2. Moreover, we confirmed by GSEA analysis that these pathways were significantly enriched in the PDLS while PB signature was enriched in MCL-PB samples (Fig. S2A).

BCR signature was also increased in 3 out of the 4 patients analyzed (Fig. S2B) but did not reach significance. Of note, the sample that behaves differently (MCL 10) belongs to a post-ibrutinib case at the time of relapse that may explain this outlier behavior. In fact, the down-regulation of BCR signaling after treatment with BTK inhibitors has been recently described in Richter transformation patients [35].

Furthermore, GSEA analysis using canonical pathways [36] uncovered that B cells from PDLS, compared to MCL-PB, exhibited an overrepresentation of relevant pathways in MCL pathogenesis including two fundamental blocks. The first one, composed of proliferation (E2F, MYC, KRAS), survival (NF-kB, TNF), metabolic pathways (OXPHOS, glucose and amino acid metabolism), “housekeeping” cellular processes (protein and RNA synthesis), DNA damage/repair, altogether reflecting the active state of these MCL tumoroids. The second block was composed of Immune pathways including activation, antigen presentation together with cytokines and chemokines fundamental for a LN-like immune microenvironment (Figs. 2D and S2C and Tables S3 and S4).

**Fig. 3:**
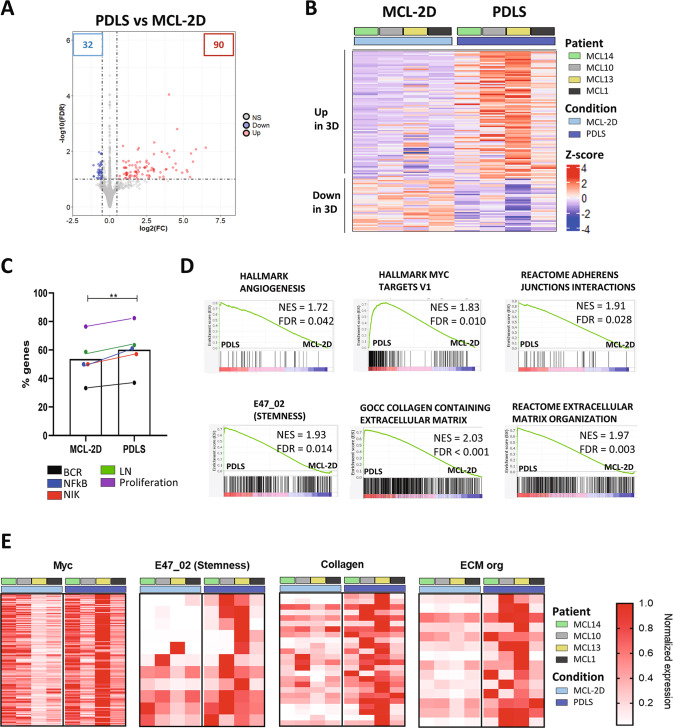
PDLS transcriptome compared to MCL-2D. **A** Volcano plot representing the differentially expressed genes (DEG) between MCL-PDLS after 7 days of culture to 2D-MCL culture with monocytes and cytokines. DEG were obtained by a paired (*n* = 4) DESeq2 analysis (FDR < 0.1 and absolute log_2_FC > 0.5). **B** Heatmap of DEG for the individual patients (*n* = 4). **C** Percentage of genes described in each pathway (as in Fig. 2) [12, 34] which are upregulated in the PDLS or in MCL-2D. **D** GSEA plots representing significantly enriched pathways (FDR < 0.05) in the PDLS compared to 2D-MCL. **E** Heatmaps of leading genes of the indicated gene sets in (**D**).

Moreover, we confirmed that the optimized culture conditions for primary MCL cells in 3D (PDLS) were superior to a conventional 2D approach including the same cytokine cocktail and monocytes. In this regard, differential expression analysis of 3D (PDLS) vs. 2D (MCL-2D) approaches allowed the identification of 90 genes upregulated in the PDLS condition, while only 32 genes were increased in the 2D culture (Fig. 3A, B). Noteworthy, PDLS was superior than MCL-2D in recapitulating MCL hallmark pathways (BCR, NF-kB, NIK and proliferation) (Fig. 3C). Moreover, GSEA highlighted additional pathways upregulated in PDLS compared to MCL-2D including angiogenesis, cell cycle, oncogene activation (KRAS and MYC), cell adhesion, stemness, post-translational modification (O-glycosylation) and extracellular matrix (ECM) involvement (Table S5 and Fig. 3D). The leading-edge genes of representative signatures from Fig. 3C are presented in heatmaps (Fig. 3E) and gene names are included in Table S6. In this regard, it is noteworthy the reorganization of the ECM that occurs in 3D including the upregulation of several collagens (*COL12A1*, *COL22A1*, *COL6A3* and *COL7A1*), the immunosuppressive tenascin (*TNC*) together with metalloproteinases (*MMP9*, *MMP16* and *MMP19*) (Table S6).

Altogether, these results support that MCL-PDLS represent a robust 3D model recapitulating fundamental biological pathways of MCL in secondary lymphoid organs such as the LN.

### Patient-derived MCL spheroids exhibit a T-cell immune exhaustion profile reminiscent of MCL-LN

**Fig. 4:**
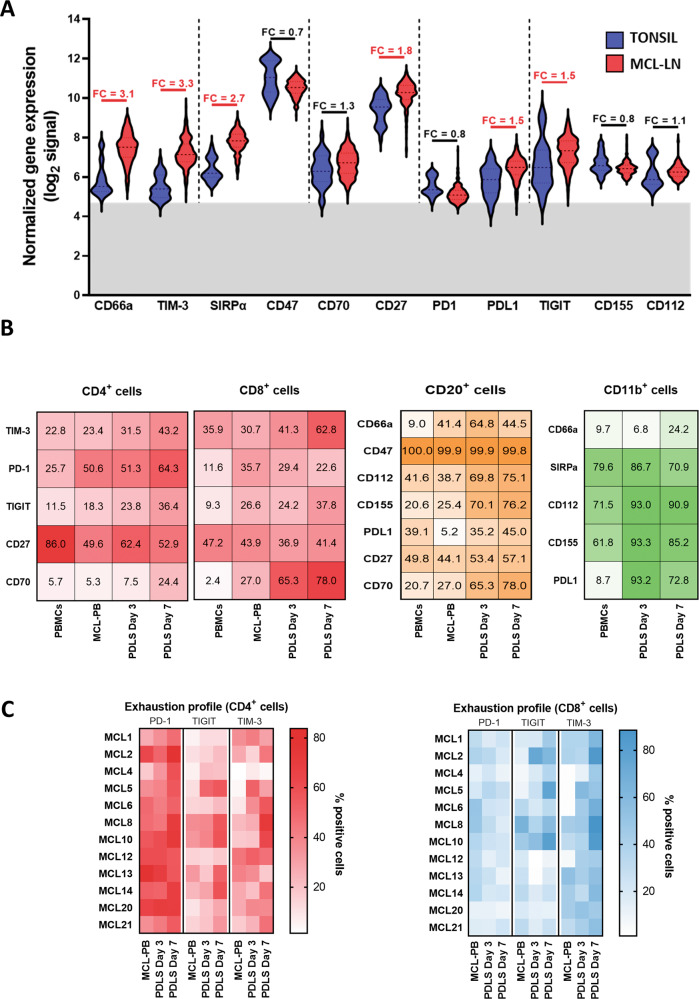
Evolution of the immune profile in the MCL-PDLS. **A** Differential gene expression analysis from microarray data obtained from public repositories (detailed in Supplementary Methods) showed upregulation of several immune regulators in MCL-LN (*n* = 199) compared to a normal tonsil (*n* = 30). In red those comparatives that are statistically significant (*p* value <0.001 and absolute FC > 1.5). **B** Percentage of positive cells assessed by flow cytometry for the immune regulators represented in (**A**) in B, T cells and monocytes. Data are represented as mean values after thawing (MCL-PB or monocytes) or in the PDLS after 3 and 7 days of culture. PBMCs from healthy donors were included as reference. **C** Expression levels of immune exhaustion markers in CD4^+^ and CD8^+^ T cells in each individual patient in the same experimental conditions as in (**B**). Data are represented as percentage of positive cells.

It has been described that MCL exhibits features of exhaustion, including high expression of PD-1 and TIGIT in both CD4 and CD8 T cells [22], as well as the presence of the corresponding ligands PD-L1, CD155 and CD112 in lymphoma cells and/or macrophages. By means of a bioinformatics analyses of public databases we compared the expression of a wide panel of immune checkpoints and its ligands in normal tonsils and in LN from MCL patients (MCL-LN), and we confirmed those published results and identified additional immune regulators overexpressed in MCL-LN. Among the receptor-ligand pairs analyzed, we highlight the increase in RNA levels of CD66a and TIM3, SIRPα, CD27, together with the already known PD-L1 and TIGIT (Fig. 4A). We next sought to determine if MCL-PDLS recapitulate this immune exhaustion profile by flow cytometry analysis of these immune regulators and their ligands, including those whose expression levels were not significantly different between MCL and normal tonsils. We performed this analysis in the PB sample just after thawing (MCL-PB) and in PDLS after 3- and 7-days culture. Moreover, we added a control of PBMCs from healthy donors. Likewise, in the case of monocytes, we compared the immune regulators expression before their inclusion in the PDLS, and after being in the PDLS for 3 or 7 days. We observed that MCL-PB profile is quite similar to PBMCs control for the expression of many immune regulators in CD4 or CD8 T cells and B cells. After the PDLS culture, there was an increase of most immune checkpoints and their ligands including: TIM-3 and TIGIT in both CD4 and CD8 cells, CD70 in CD8 and PD-1 in CD4. SIRPα, CD27 and CD47 expression was basically maintained. The increase of PD-L1, CD112 and CD155 was also significant in both B (CD20+) cells and monocytes/macrophages (CD11b+) in the PDLS (Figs. 4B and S3) compared to MCL-PB. As expected, the level of immune exhaustion generally increases with the days of culture. The heatmap in Fig. 4C illustrates this phenomenon for PD-1, TIM-3 and TIGIT and also shows the interpatient variability.

Overall, we can conclude that MCL-PDLS generated from PB recapitulate the immune exhaustion features of MCL-LN and may represent a good tool for immune-oncology studies.

### Patient-derived MCL spheroids recapitulate in vivo response to ibrutinib treatment

BTK inhibitors as ibrutinib represent the standard of care to treat relapsed MCL, and is currently moving to frontline combined with standard first-line therapy (NCT02858258). Thus, we sought to determine if we could recapitulate clinical responses to ibrutinib in the MCL-PDLS system. First, we checked if the inhibitor was active in the PDLS. As shown in Fig. S4A, ibrutinib decreases tumor burden significantly, almost 50% on average albeit with interpatient variability (*n* = 17). This depletion was associated in the sensitive cases with a decrease in tumor cell proliferation and viability induced by ibrutinib (Fig. S4B, C).

**Fig. 5:**
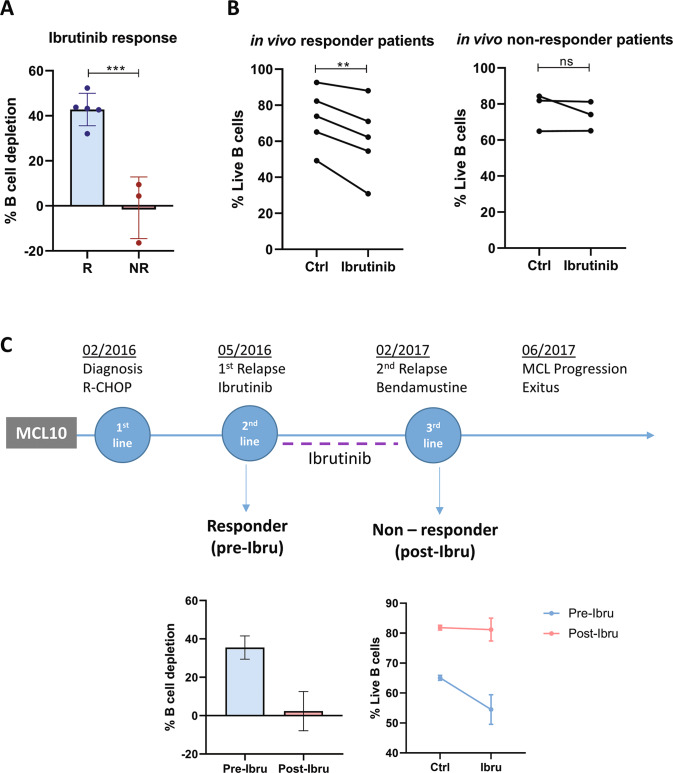
MCL-PDLS reproduces in vivo response to ibrutinib. **A** Tumor B-cell depletion after 72 h of ibrutinib treatment compared to untreated condition in PDLS generated from MCL patients who in vivo responded to ibrutinib (R) or patients who did not respond to the drug (NR). **B** B-cell viability in untreated (Ctrl) or after in vitro ibrutinib treatment (72 h) in PDLS from in vivo responder or non-responder patients. **C** Clinical case of MCL10 including timeline with the different lines of treatment. Graphs showed B-cell depletion and viability of PDLS after 72 h of treatment with ibrutinib refereed to untreated control.

Then, we generated MCL-PDLS with samples from patients who received the drug at our institution and thus can be classified into responder patients (including partial response) and non-responder patients. After 3 days, MCL-PDLS were treated with ibrutinib or not (control condition) and cell count and viability were determined by flow cytometry. Noteworthy, MCL-PDLS reproduced with high degree of accuracy the in vivo response (Fig. 5A), and only the PDLS derived from ibrutinib-responder patients showed a decrease in their viability in B-cell fraction when adding ibrutinib (Fig. 5B).

Interestingly, one of the patients included in this study, MCL10, received ibrutinib as 2nd line treatment after relapsing from R-CHOP initial treatment. At first, MCL10 was responsive to ibrutinib achieving a partial response, followed by a new progression 9 months later. We were able to generate PDLS from MCL10 from the sensitive pre-ibrutinib sample and with the samples of the second relapse, post-ibrutinib, when the patient was longer responding to ibrutinib. PDLS recapitulate this in vivo scenario faithfully, as displayed in Fig. 5C. Ibrutinib induced more than 30% B-cell depletion in the PDLS from the first relapse that initially responded to ibrutinib, while no effect was seen when treating the PDLS from ibrutinib progression. Similar results were obtained when viability was assessed in these two PDLS (Fig. 5C).

These results support the PDLS model as a robust system to predict the response to BTK inhibitors.

### Patient-derived MCL spheroids engage immune activation in response to immune checkpoint inhibitors

**Fig. 6:**
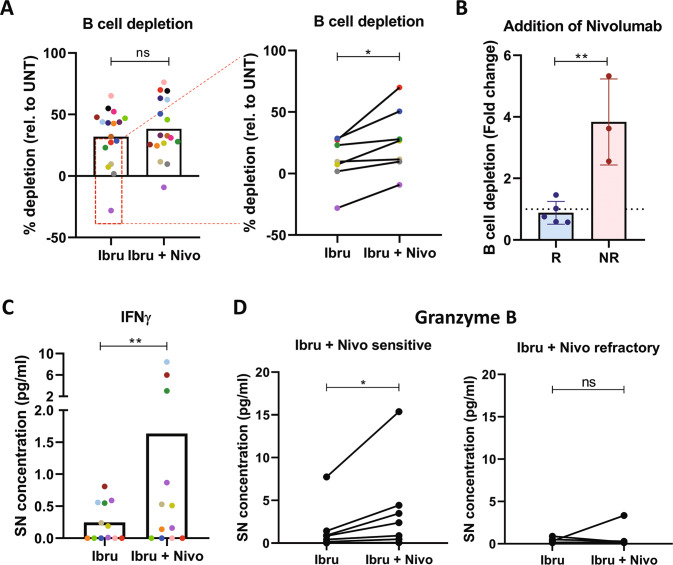
Ibrutinib and nivolumab combination is effective in ibrutinib-resistant patients by activating the immune system. **A** B-cell depletion of ibrutinib and nivolumab combination (Ibru + Nivo) compared to ibrutinib monotherapy (Ibru) (*n* = 17), with a significant benefit in most resistant patients (*n* = 7). **B** Effect of adding nivolumab to ibrutinib treatment in in vivo responder (R) or non-responder (NR) patients, represented as the fold change of B-cell depletion induced by the combination compared to Ibrutinib monotherapy. **C** Interferon gamma (IFNγ) concentration in PDLS supernatants comparing ibrutinib monotherapy or in combination with Nivolumab in MCL-PDLS. **D** Granzyme B secretion comparing ibrutinib monotherapy with ibrutinib and nivolumab combo, in sensitive or resistant patients to the combination. Cytometric Bead Array (CBA) analysis of cell culture supernatants was used in (**C**) and (**D**) (*n* = 12).

Ibrutinib is known to be an effective drug for MCL treatment, but most patients acquire resistance and eventually relapse. Therefore, effective therapeutic alternatives represent an unmet clinical need for MCL. In this scenario, the combination of ibrutinib with the anti-PD1 nivolumab has been studied in clinical trials [37] in several types of NHL but not in MCL, showing good results in CLL Richter transformation [38]. Thus, we analyzed the efficacy of this combination compared to ibrutinib in the PDLS system. As shown in Fig. 6A, B-cell depletion induced by the combination was slightly higher than ibrutinib monotherapy, but without reaching statistical significance. However, we noticed that those MCL cases with limited responses to ibrutinib (B-cell depletion below the mean (31.9%)), were those that benefit most from the combination, and B-cell depletion was significantly superior than ibrutinib alone (Fig. 6A, right).

Furthermore, in vivo non-responder patients to ibrutinib achieved a higher B-cell depletion with nivolumab combination, while the in vivo responder patients did not benefit from nivolumab addition in vitro (Fig. 6B).

Next, we classified our patients according to *TP53* status between wild-type and mutated (Table 1). Interestingly, addition of nivolumab only benefited those cases who did not have altered *TP53*, while patients who carried mutations or deletions showed a similar B-cell depletion (fold change = 1) when comparing the combination or ibrutinib as monotherapy (Fig. S5A).

Finally, we investigated whether addition of nivolumab activated the immune system toward an anti-tumoral response by analyzing the release of IFNγ and Granzyme B in the MCL-PDLS supernatants as a read-out of immune activation. Interestingly, the combination led to significant higher IFNγ concentrations in PDLS supernatants compared to ibrutinib alone, suggesting that the MCL-PDLS system may engage a Th1 anti-tumoral response (Fig. 6C). Granzyme B levels were also increased but without reaching statistical significance (Fig. S5B). However, when patients were classified into sensitive or refractory to the combination, according to the in vitro response (fold change depletion (ibru + nivo vs. ibru) >1.2), we observed the increase of granzyme B levels in supernatants from sensitive PDLS, while not in those from refractory PDLS (Fig. 6D). Likewise, the percentage of CD8 T cells increased in PDLS sensitive to the combination (Fig. S5C).

Thus, we found evidence that a cytotoxic response (Granzyme B release) is activated by the addition of nivolumab to the PDLS system and it is associated to the efficacy of the combination.

## Discussion

B lymphoma mainly develop within LN as aggregates of tumor cells densely packed with their surrounding microenvironment, creating a tumor specific niche. In the precise case of LN-resident MCL cells, they rely mostly on BCR-mediated signaling and NF-kB pathways and have therefore a clear role in proliferation of LN-MCL cells [12]. These signaling pathways are the results of MCL crosstalk with the TME in the LN, mainly T cells, macrophages and resident stromal cells as FDCs. In order to recapitulate these complex interactions in vitro, patient-derived 2D co-cultures supplemented with specific cytokines and growth factor cocktails have been established [17]. However, it is currently accepted that 3D models better represent cancer biology, signaling pathways [39, 40], and specially B and T-cell activation, as they are influenced by physical forces that are not recapitulated in 2D cultures [41]. Thus, in the era of precision medicine, it is mandatory to establish robust and reproducible patient-derived 3D systems. This is even more urgent in a rare and heterogeneous disease as MCL, where preclinical efficacy of novel agents and combinations will ease the design of clinical trials where patient recruitment is always challenging.

For all these reasons, we endeavored to set the first patient-derived MCL lymphoma 3D system as a real alternative to costly Patient-Derived Xenograft (PDX) model. MCL-PDX have been successfully established in this disease and have proven to be useful for antibody therapy [42–44]. However, they do not represent the best option for large screenings and do not recapitulate a human microenvironment, unless using humanized (hu-PDX) mice, which elevates the cost and complicates the design. Thus, one can envision PDX and hu-PDX as a last step of validation before clinical translation.

MCL-PDLS represents an affordable and robust system for a number of reasons:

First, both tumor B cells and autologous T cells maintain good viability and engage proliferation for at least 1-week, a window which allows to analyze the efficacy of most therapeutic agents. It is fundamental to consider the myeloid compartment as a part of the MCL niche, disease pathogenesis and a source of immunosuppressive signals [20, 21, 45]. As the percentage of autologous monocytes in the original PB sample was extremely low due to tumor B-cell expansion [46], we decided to introduce monocytes from healthy donors in a ratio that reflects macrophage infiltration in MCL biopsies [31].

Second, in this study most of the samples were PBMCs from PB, both from cMCL (*n* = 10) and nnMCL (*n* = 8). PB samples represent the most common and abundant material available as a high proportion of MCL patients present with leukemic disease [47]. Thanks to the optimized culture conditions, PDLS fairly recapitulate MCL-LN signature and fundamental hallmarks as NF-kB, BCR and proliferation signature. This is of special interest considering the scarce availability of MCL-LN samples. Moreover, additional relevant pathways identified include metabolic pathways (OXPHOS [48], glucose and amino acids metabolism) and housekeeping processes (protein/RNA synthesis), reflecting that MCL-PDLS are a living and dynamic system. In addition, despite the absence of external additional of ECM in the system, we were able to demonstrate that the 3D conformation together with monocytes that differentiate into macrophages in the MCL-PDLS, favor the generation of ECM components including several types collagen (*COL12A1*, *COL22A1*, *COL6A3* and *COL7A1*), the immunosuppressive tenascin (*TNC*) together with metalloproteinases (*MMP9*, *MMP16* and *MMP19*), creating a more fibrotic TME typical of M2-like macrophages [49, 50].

Third, MCL-PDLS recapitulate the immune TME exhaustion features of MCL-LN [51] and may represent a good tool for immune-oncology studies. It is noteworthy the overrepresentation of gene sets related to immune pathways identified by RNA-seq (Tables S3 and S4). Thus, PDLS may be useful to test bi-specific antibodies and T-cell engagers due to the presence of autologous T-cells. Moreover, this MCL-PDLS has the advantage of including myeloid immunosuppressive cells expressing key ICP ligands such as PDL1, CD66a, SIRPα and the TIGIT ligands CD112 and CD155.

Fourth, PDLS recapitulate in vivo responses to biological agents targeting tumor cells and TME such as ibrutinib, and represents a platform to study novel combination of BTK inhibitors. Ibrutinib is approved and very active in relapse/refractory (R/R) MCL [52], but most patients eventually develop resistance. Thus, second and third generation BTKi have been developed and will offer an advantage in certain settings [53]. Likewise, new combinatorial approaches have been investigated, including anti-CD20 mAb [54] and/or BH3 mimetics [55, 56]. In this context we propose the combination with the anti-PD1 mAb nivolumab specifically for those patients with limited ibrutinib responses. This has been analyzed in the phase I/IIa LYM1002 study (NCT02329847) for several relapsed/refractory B-cell malignancies. The best responses (>60%) were obtained for chronic lymphocytic leukemia (CLL) and Richter Syndrome [57], and current efforts are driven toward the identification of potential biomarkers of response to identify beforehand those patients who will benefit from this combination [58]. Likewise, the preliminary efficacy of the anti-PD1 pembrolizumab in combination with ibrutinib is under investigation in a phase I/IIa trial (NCT03153202) in R/R CLL and R/R MCL. Thus, we envision our MCL-PDLS system as a complementary in vitro tool for phase 1/2 trials to help identifying biomarkers of response and mechanisms of resistance.

In summary, MCL-PDLS represents a novel 3D model maintaining fundamental hallmarks of MCL-LN, that may serve a platform to perform preclinical screening of novel targeted therapies, immunotherapies and cell therapies in a robust, 96-well format and affordable patient-derived 3D system. Future perspectives we are approaching to improve and complexify these systems include: the integration of ECM and relevant stromal cells (FDC), and the inclusion of these tumoroids in a microvascularized system.

## Supplementary information

Supplemental material

Supp video 1

Supp video 2

## Data Availability

RNA sequencing data generated and analyzed during the current study are available in the European Genome-phenome Archive (http://ebi.ac.uk/ega/) under accession number EGAS00001006964. The datasets generated and/or analyzed during the current study are available from the corresponding author on reasonable request.
