# Supplementary material for: A novel patient-derived 3D model recapitulates mantle cell lymphoma lymph node signaling, immune profile and in vivo ibrutinib responses
Source: Leukemia. 2023 Apr 8;37(6):1311–23. doi: 10.1038/s41375-023-01885-1 (PMC10244172; doi:10.1038/s41375-023-01885-1)
Supplement: Supplementary file 1 — Supplemental material [file 41375_2023_1885_MOESM1_ESM.pdf]

SUPPLEMENTAL METHODS

Patient samples and healthy donor peripheral blood mononuclear cells

MCL primary samples used in this study belong to Hematopathology collection, registered in the Biobank of IDIBAPS-Hospital Clínic, Barcelona (R121004-094) and in the National Registry of Biobanks-ISCIII (C.0000397), according to *Real Decreto 1716/2011*. This collection corresponds to biological material remaining from the samples used for the diagnosis, according to the World Health Organization (WHO), in the Hematopathology Unit of Hospital Clinic de Barcelona (HCB, Barcelona, Spain). Informed consent has been obtained from each patient in accordance with the Institutional Ethics Committee of the HCB and the Declaration of Helsinki.

Peripheral blood mononuclear cells (PBMCs) were obtained from healthy donor buffy coats provided by Banc de Sang i Teixits (Barcelona, Spain) after Ficoll-Paque gradient separation (GE Healthcare, Chicago, IL, USA). Monocytes were purified using $CD14^+$ magnetic beads and LS columns (Miltenyi Biotec, Bergisch Gladbach, Germany) and purity >98% was verified by flow cytometry (FACS Fortessa (BD Biosciences, Franklin Lanes, NJ, USA)). Monocytes were cryopreserved with 10% DMSO (Sigma-Aldrich, St. Louis, MO, USA) and stored in liquid nitrogen until use at IDIBAPS Biobank.

MCL-PDLS imaging

PDLS formation and growth were monitored by brightfield illumination on the automated digital microscope Cytation 1 (Biotek, Agilent, Santa Clara, CA, USA) under temperature (37ºC) and CO_2/O_2 gas control. Z-stacking function (n=5) was always performed to account for the third dimension. Image analysis and Z-projection were done with manufacturer´s software Gen 5 (Biotek).

PDLS were also characterized by Selective Plane Illumination Microscopy (SPIM). Day 7 PDLS were fixed in 4% paraformaldehyde (PFA) for 12h, then PFA was diluted to 0.5% with PBS after several rinse cycles, and finally replaced by blocking/permeabilization buffer (PBS + 2% FBS + 2% BSA + 0.6% Triton + 0.01% Azide) and incubated overnight (ON) with agitation at room temperature (RT). PDLS were then labeled with 10 µg/mL propidium iodide (Thermo Fisher Scientific) and incubated for 4h at RT with agitation, washed and included in agarose.

Then, PDLS included in agarose were cleared using methanol and Benzyl Alcohol/Benzyl Benzoate (BABB) reagent. Finally, PDLS included were imaged by SPIM microscope (ZEISS Lightsheet Z.7, Imactiv 3D, Toulouse) and image-processing algorithms were developed in MATLAB.

Monocyte-Macrophage differentiation and polarization analysis

To assess MCL induced monocyte-macrophage differentiation /polarization, CD11b$^+$ Far Red$^+$ cells were recovered by cells sorting (FACS Aria, BD) from PDLS (day 7), and RNA isolated with TRIZol following manufacturer's protocol (Thermo Fisher Scientific). cDNA was synthetized using Preamp RT Master Mix (Fluidigm, San Francisco, CA, USA). Next, cDNA was pre-amplified for the following genes: *CXCL11, CCL5, MRC1, CCL22, PMAIP1, RSG2, GUSB, ACTB, B2M* using pre-designed TaqMan probes (Thermo Scientific) and PreAmp Master Mix (Fluidigm).

Quantitative real-time PCR (qPCR) was then performed on a StepOne Real-Time PCR System (Thermo Fisher Scientific) using the same probes as the preamplification. Samples were analyzed in duplicate and expression was normalized using the mean of the three endogenous genes: *GUSB, ACTB, B2M*.

To evaluate the degree of M1/M2 polarization MCL-macrophages were compared with M1 or M2-polarized macrophages generated as follows: monocytes obtained from PBMCs as previously described were seeded in 6-wells plates at $0.5x10^6$ cells/mL in enriched medium supplemented with macrophage colony stimulating factor (M-CSF) (ThermoFisher Scientific) at 100 ng/mL. After 6 days, additional stimuli were provided to obtain M1-polarized macrophages ((20ng/mL IFNγ (Gibco, Thermo Fisher Scientific) + 100ng/mL LPS (Sigma-Aldrich)) or M2-polarized macrophages (20ng/mL IL-4 (PeproTech, Rocky Hill, NJ)) for 24h.

MCL-PDLS immune profile and activation

MCL-PDLS (day 3 and day 7) immune profile were both characterized with several panels for B (CD20+) cells, T (CD4+ and CD8+) cells and monocytes/macrophages (CD11b+) using the following antibodies PD1, TIGIT, TIM-3, CD27, CD70, CD66a, CD112, CD155, PD-L1, CD47, SIRPα followed by flow cytometry analysis. PBMCs from healthy donors were used as controls. A complete list of antibodies is provided in supplemental table S1.

Granzyme B and interferon (IFN)-γ were used as read-out of T cell activation and were analyzed in cell culture supernatants using Cytometric Bead Array (CBA) kits and FACS Fortessa (BD Biosciences) following manufacturer´s instructions. Data was analyzed using FCAP Array^{TM} v.3.0 Software (BD Biosciences).

RNA-seq and data analysis

Day 7 PDLS was disaggregated, labelled with LIVE/DEAD Aqua, CD20 - eIF450 (Thermo Fisher Scientific) and CD3-FITC (BD Biosciences). CD20$^+$, CD3$^-$ Aqua$^-$ cells were recovered using a BD FACSARIA II sorter (Cytometry and cell sorting facility, IDIBAPS) and RNA extracted as before. Likewise, CD20+, CD3- Aqua- cells isolated from the original MCL sample at thawing (day 0) were used as a comparator for RNA-seq studies.

RNA was assayed for quantity and quality using Qubit RNA HS Assay (Life Technologies) and RNA 6000 Nano Assay on a Bioanalyzer 2100. Stranded RNA-seq libraries were performed for 150 ng of mRNA using the TruSeq library kit (Illumina, San Diego, CA, USA). Libraries were sequenced on a NextSeq2000 (Illumina) in a 2x50bp length with a coverage of >40 million paired-reads per simple.

Sequencing reads were trimmed using trimmomatic (version 0.38),[1] and ribosomic RNA reads were filtered out using SortMeRNA (version 2.1b).[2] Gene-level counts (GRCh38.p13; Ensembl release 100) were calculated using kallisto (version 0.46.1)[3] and tximport (version 1.6.0).[4] Differential expression was conducted using DESeq2 (version 1.18.1).[5] Shrinkage of effect size was performed using the apeglm method.[6] Adjusted *P* value (*Q*) <0.10 and absolute log2-transformed fold change >0.5 were used to identify differentially expressed genes (DEGs).

Gene-set enrichment analyses (GSEAs) were conducted with GSEA software (version 4.2.3), [7] using the pre-ranked modality and log2FC results obtained from DESeq2 as input data. Hallmark gene sets, the C2 curated gene sets, the C3 motif gene sets, the C5 gene ontology gene sets (Molecular Signature Database v7.5), and custom gene sets [8,9] were used. Heatmaps of selected genes were generated using GraphPad software. RNA-seq data have been deposited at the European Genome-phenome Archive (accession number EGAS00001006964) and will be publicly accessible at the time of publication.

Metadata comparative analysis

mRNA relative expression levels of selected immune checkpoints in whole lysates of Mantle Cell Lymphoma lymph nodes (MCL-LN, n=199) was compared with normal tonsils (TONSIL n=30) according to GEP public databases (GSE21452, GSE93291, GSE70910, GSE70927, GSE132929, GSE3526, GSE7307, GSE39503, GSE43346, GSE65135, GSE65136 and GSE71810). These selected data were all generated with Affymetrix Human Genome U133 Plus 2.0. Briefly, CEL files were normalized using the Expression Console ™ Software v1.4.1.46 (Affymetrix). To take in consideration the batch effect, join data were normalized using the Limma package included in Transcriptome Analysis console (Applied Biosystems).

Statistical analysis

Data were analyzed using Prism Software 8.0 (GraphPad Software, San Diego, CA, USA). Depending on the assay and based on Shapiro-Wilk normality test, paired t test or Wilcoxon test were used. Data were represented as the mean values of the patients. Differences between the results of comparative tests were

considered significant if the two-sided P value was less than 0.05. The statistical significance convention used along the manuscript was as follows: * p<0.05, **p<0.01, ***p<0.001 and ****p<0.0001.

Bibliography

1 Bolger AM, Lohse M, Usadel B. Trimmomatic: a flexible trimmer for Illumina sequence data. *Bioinformatics* 2014; **30**: 2114–2120.

2 Kopylova E, Noé L, Touzet H. SortMeRNA: fast and accurate filtering of ribosomal RNAs in metatranscriptomic data. *Bioinformatics* 2012; **28**: 3211–3217.

3 Bray NL, Pimentel H, Melsted P, Pachter L. Near-optimal probabilistic RNA-seq quantification. *Nat Biotechnol* 2016; **34**: 525–527.

4 Soneson C, Love MI, Robinson MD. Differential analyses for RNA-seq: transcript-level estimates improve gene-level inferences. *F1000Research* 2015; **4**: 1521.

5 Love MI, Huber W, Anders S. Moderated estimation of fold change and dispersion for RNA-seq data with DESeq2. *Genome Biol* 2014; **15**: 550.

6 Zhu A, Ibrahim JG, Love MI. Heavy-tailed prior distributions for sequence count data: removing the noise and preserving large differences. *Bioinformatics* 2019; **35**: 2084–2092.

7 Subramanian A, Kuehn H, Gould J, Tamayo P, Mesirov JP. GSEA-P: a desktop application for Gene Set Enrichment Analysis. *Bioinformatics* 2007; **23**: 3251–3253.

8 Saba NS, Liu D, Herman SEMM, Underbayev C, Tian X, Behrend D *et al.* Pathogenic role of B-cell receptor signaling and canonical NF-κB activation in mantle cell lymphoma. *Blood* 2016; **128**: 82–92.

9 Rosenwald A, Wright G, Wiestner A, Chan WC, Connors JM, Campo E *et al.* The proliferation gene expression signature is a quantitative integrator of oncogenic events that predicts survival in mantle cell lymphoma. *Cancer Cell* 2003; **3**: 185–197.

SUPPLEMENTARY FIGURES

Figure S1

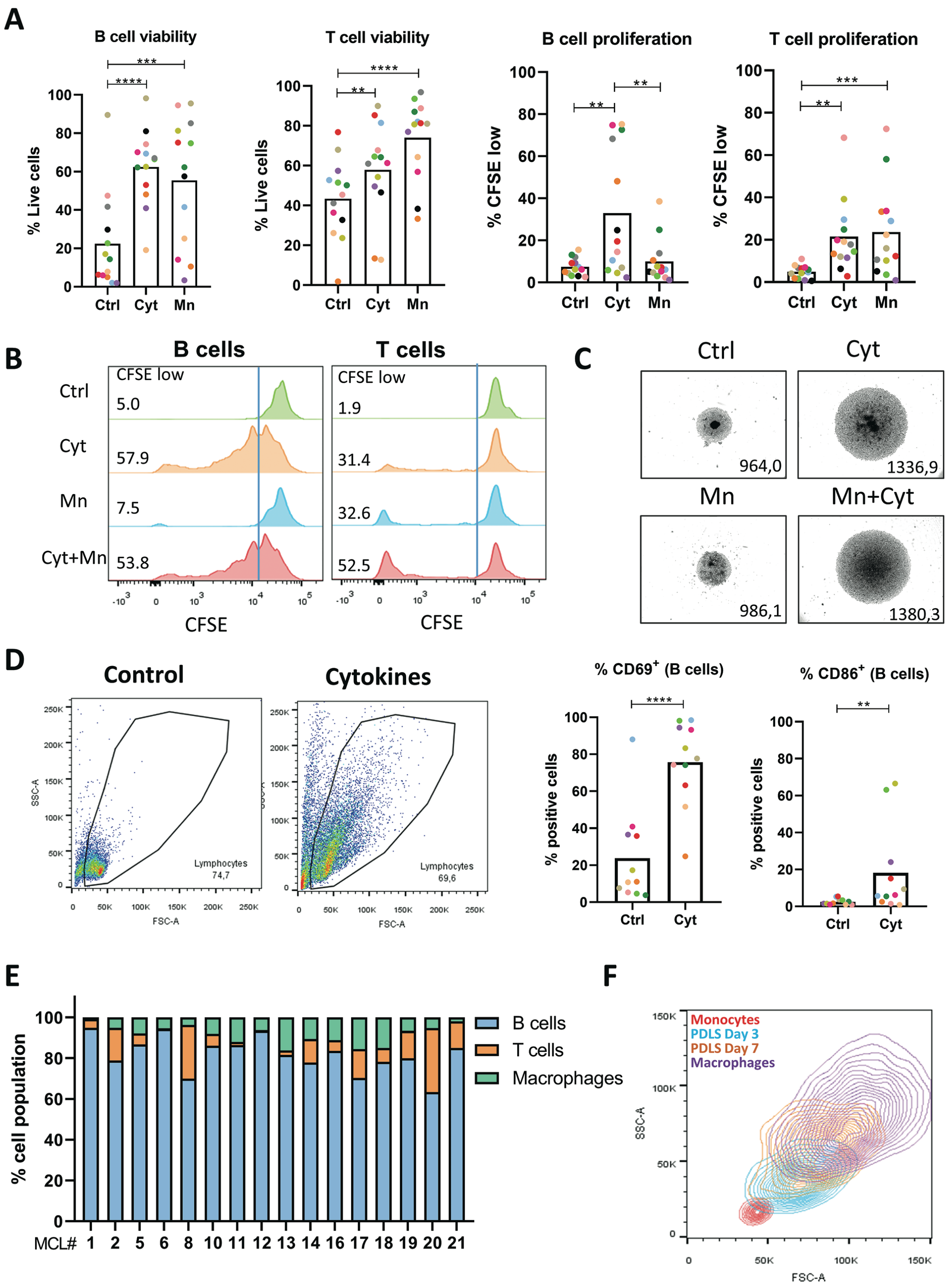

Figure S1. Effect of cytokines and/or monocytes on PDLS culture. **(A)** B/T cell viability and proliferation (%CFSE low) of MCL-PDLS cultured during 7 days with cytokines (Cyt) or monocytes (Mn) compared to the control (Ctrl) condition of non-stimulated PDLS. **(B)** Representative CFSE histogram (MCL10) plot showing B- and T cell proliferation when adding cytokines and/or monocytes. Percentage of CFSE low cells is indicated. **(C)** PDLS (MCL13) brightfield imaging (Cytation 1, x40 magnification) with cytokines (Cyt) and/or monocytes (Mn) stimuli compared to control (Ctrl) condition. Size in μm is indicated**(D)** Density plots illustrates the increase in cell growth (FSC-A) and cell complexity (SSC-A) of PDLS cultured with only cytokines for 7 days. B cell activation was assessed by the percentage of CD69 and CD86 positive cells. **(E)** B, T and monocyte distribution after 7 days with cytokines and monocytes. **(F)** Evolution in size (FSC-A) and complexity (SSC-A) of CD11b+ isolated from the PDLS (MCL13) after 3 or 7 days in PDLS media, compared to monocytes or to macrophages differentiated in 2D with M-CSF.

Figure S2

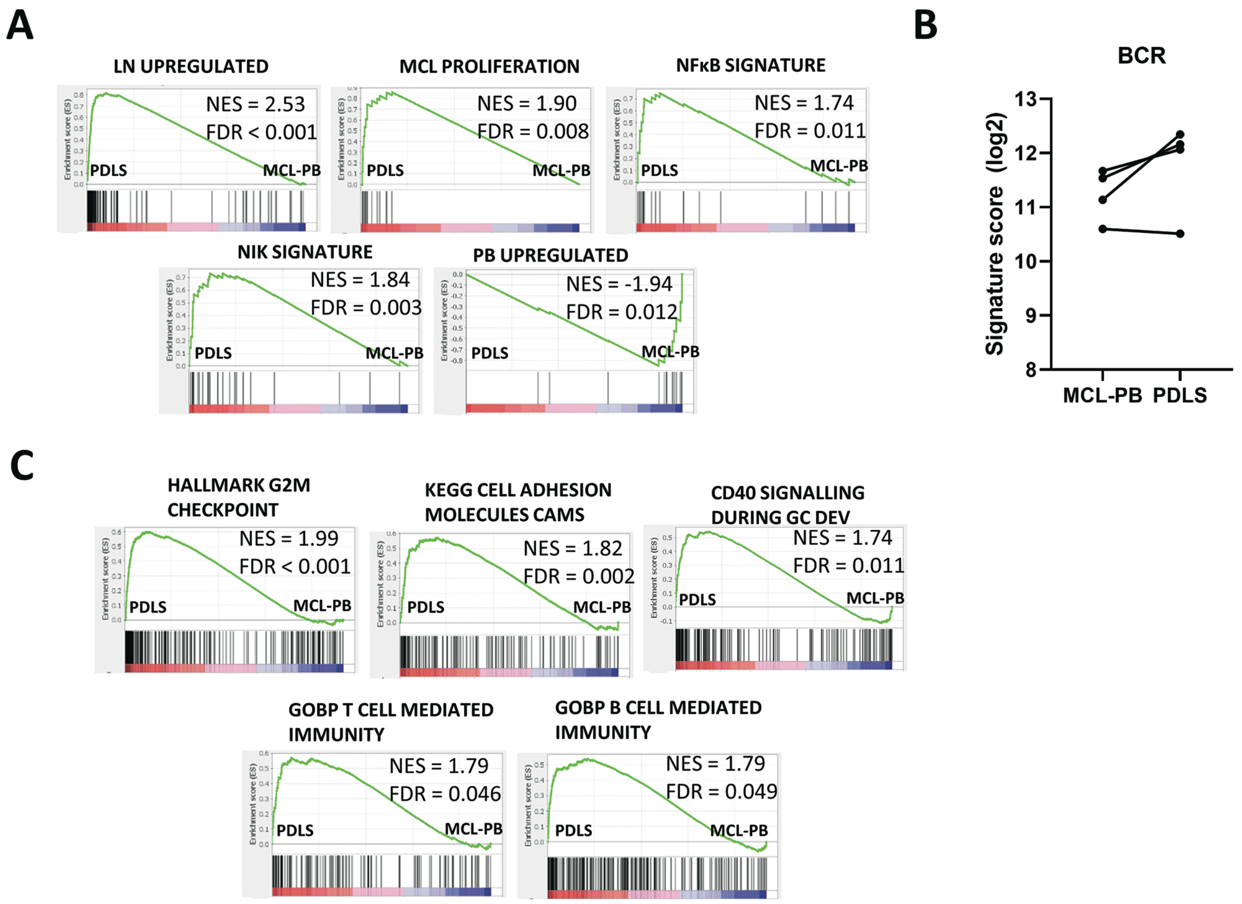

Figure S2. MCL-PDLS GSEA analysis. (A) GSEA enrichment plots representing relevant pathways in MCL pathogenesis, with significant increase or decrease in the signature score (Figure 2C). **(B)** BCR signature score calculated as explained in Figure 2C, detailed by each individual patient. **(C)** GSEA enrichment plots representing up-regulated pathways in the PDLS, related to cell cycle, adhesion and immune system.

Figure S3

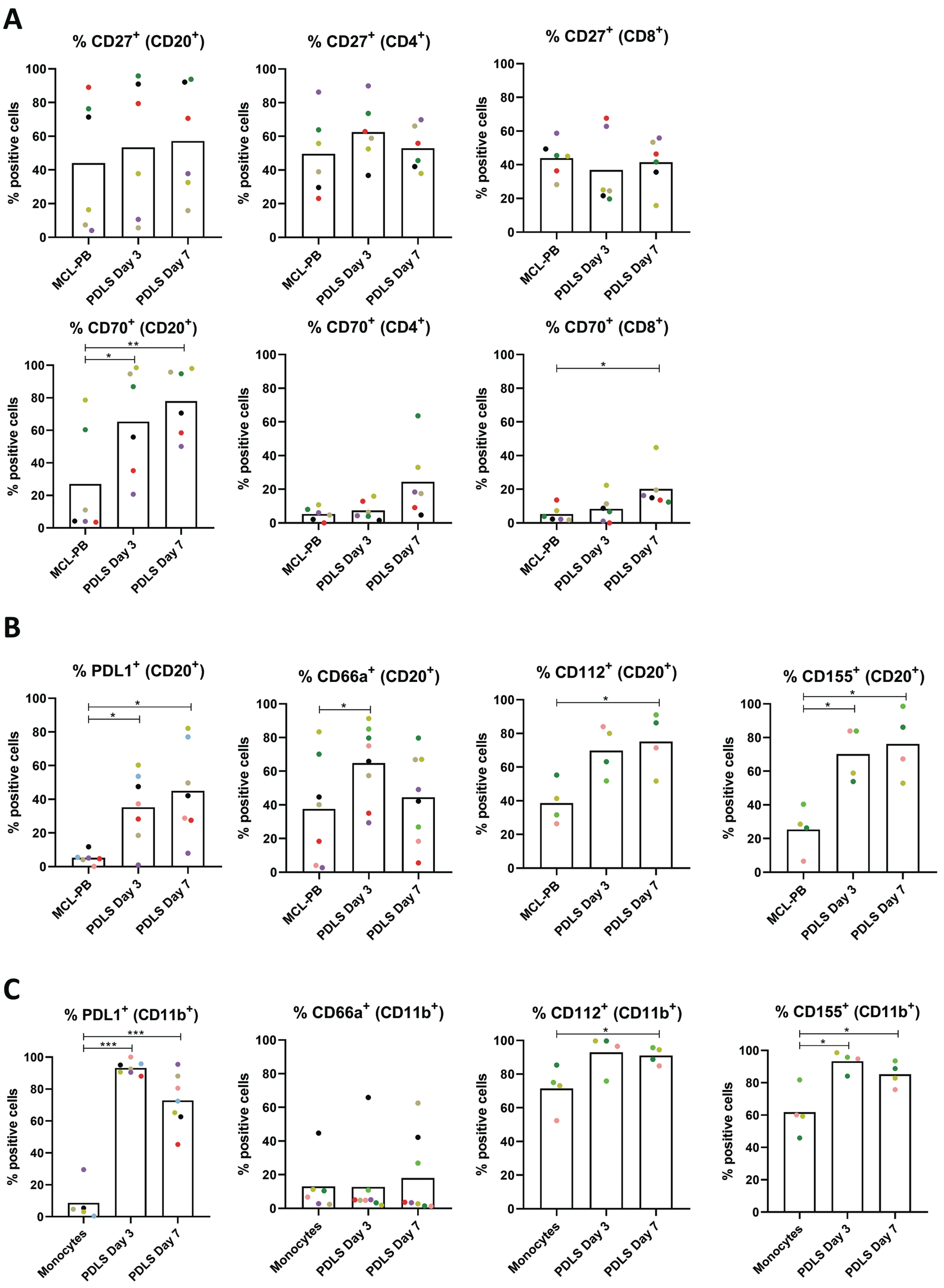

Figure S3. Up-regulation of immune modulators markers in MCL-PDLS. **(A)** Percentage of positive cells for CD70 and CD27 in B cells and CD4/CD8 T cells from MCL-PDLS (n=6) . **(B)** Percentage of positive cells of PD-L1, CD66a, CD112 and CD155 in tumor B cell population in the PB sample and from MCL-PDLS at day 3 or day 7 of culture (n=4-8). **(C)** Percentage of positive cells of ICP ligands in monocytes and macrophages from PDLS after 3 or 7 days of culture (n=4-8).

Figure S4

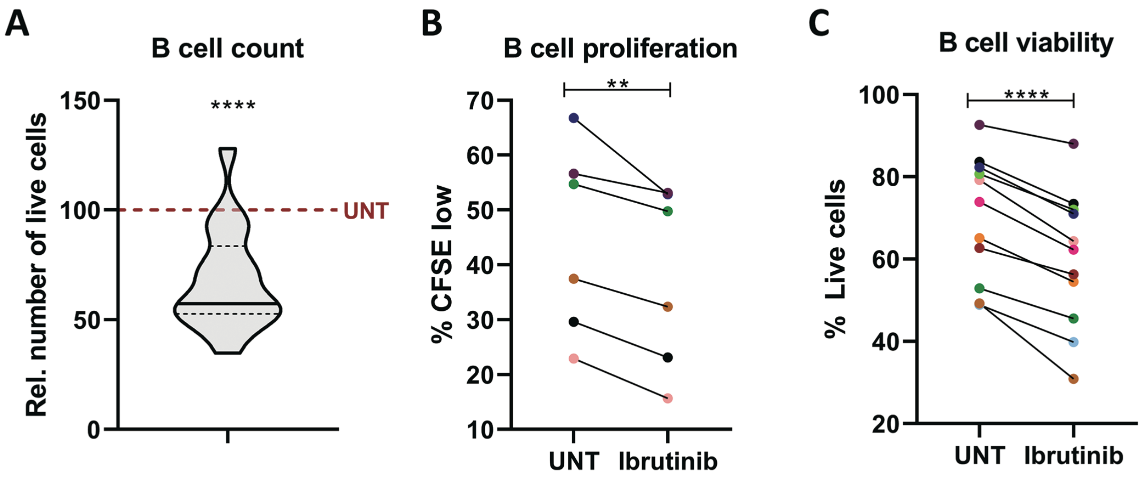

Figure S4. Ibrutinib is active in MCL-PDLS. MCL-PDLS were treated at day 3 with ibrutinib. B cell count (n=17) **(A),** B cell proliferation quantified by the percentage of CFSE low **(B)** and B cell viability measured by percentage of negative LIVE/DEAD **(C)** were assessed after 72h of ibrutinib treatment compared to untreated (UNT) samples.

Figure S5

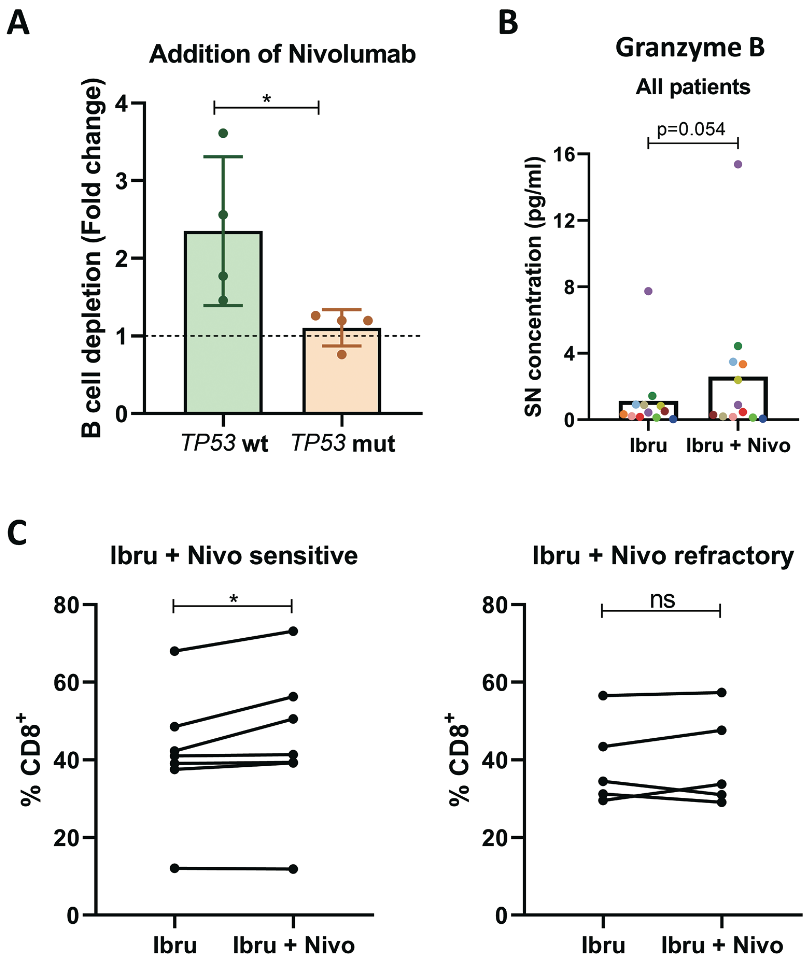

Figure S5. Ibrutinib and nivolumab combination in MCL-PDLS. (A) Effect of adding nivolumab to ibrutinib treatment in patients with *TP53* gene mutated (mut) or non-mutated (wild-type (wt)), represented as the fold change of B cell depletion. **(B)** Granzyme B concentration by CBA comparing ibrutinib monotherapy or in combination with nivolumab**. (C)** Percentage of CD8+ T cells (CD3+gated) after ibrutinib or ibrutinib +nivolumab in sensitive or resistant MCL-PDLS to the combination.

Supplemental video 1. Time-lapse video of MCL-PDLS (MCL1) evolution from D1 to D7. Images were taken every 12h in a Cytation 1 device (magnification x40).

Supplemental video 2. 3D reconstruction of MCL-PDLS (MCL1) by SPIM microscopy using MATLAB software.

SUPPLEMENTARY TABLES

Table S1. Antibodies used in flow cytometry

Target	Conjugate	Clone	Dilution
CD112	PE	R2.525	1/40
CD155	BV605	SKII.4	1/100
CD19	PE	SJ25C1	1/200
CD20	eFluor 450	2H7	1/50
CD27	PE-Cy7	LG.7F9	1/100
CD3	PE-Cy5	UCHT1	1.5/100
CD4	SB600	RPA-T4	1.5/100
CD47	PE-Cy7	B6H12	1/20
CD66a	AF488	283340	1/50
CD70	APC	REA292	1/20
CD8	APC-H7	SK1	1/100
PD-1	PE-Cy7	EH12.1	1/50
PD-L1	PE	MIH1	1/200
SIRPα	APC	15-414	1/200
TIGIT	APC	MBSA43	1/50
TIM-3	PE	F38-2E2	1/50

Table S2. Leading edge genes of specific signatures from Figure 2C

LN

ANLN
ASPM
AURKA
AURKB
BID
BUB1
BUB1B
CCL22
CDC20
CDC25A
CDC45
CDC6
CDCA3
CDCA5
CDCA8
CDK1
CDKN3
CENPA
CENPE
CENPF
CENPM
CEP55
CLSPN
CTNNAL1
DLGAP5
DTL
E2F8
ESCO2
ESPL1
EXO1
FAM72A
FEN1
FOXM1
HJURP
IPCEF1
KIF14
KIF20A
KIF23
KIF2C
KIFC1
LTBP1
MCM10
MELK
MKI67
MND1
MRPS14
NCAPG
NCAPG2
NCAPH
NEIL3
NEK2
NEURL1B
NLRP3
NME1
NUF2
PBK
PLK4
POLQ
PTPN7
RRM2
SIRPA
SKA3
SMC2
SPAG5
TOP2A
TOX
TPX2
TYMS
UBE2C
UBE2T
UHRF1

MCL Proli

AIMP2
AP3S1
APEX1
C1QBP
CBX3
CCT2
CCT3
CCT4
CCT5
CCT7
CDC20
CDC45
CDK4
CLNS1A
CNBP
COPS5
COX5A
CTPS1
DEK
EEF1B2
EIF1AX
EIF2S1
EIF2S2
EIF3B
EIF4A1
EIF4E
ERH
EXOSC7
FBL
GLO1
GNL3
GOT2
GSPT1
H2AZ1
HDAC2
HDGF
HNRNPA1
HNRNPA2B1
HNRNPA3
HNRNPC
HNRNPD
HNRNPR
HNRNPU
HPRT1
HSP90AB1
HSPD1
HSPE1
IARS1
IFRD1
ILF2
KARS1
KPNA2
LDHA
LSM7
MAD2L1
MCM2
MCM4
MCM6
MCM7
MRPL23
MRPL9
MRPS18B
NDUFAB1
NHP2
NME1
NOLC1
NOP16
NPM1
ODC1
PCNA
POLD2
POLE3
PPIA
PPM1G
PRDX4
PRPF31
PSMA1
PSMA2
PSMA4
PSMA6
PSMA7
PSMB2
PSMB3
PSMC4
PSMC6
PSMD1
PSMD14
PSMD3
PSMD7
PTGES3
PWP1
RAN
RANBP1
RFC4
RPL18
RPL34
RPL6
RPS2
RPS5
RRM1
RRP9
RUVBL2
SERBP1
SET
SF3B3
SLC25A3

SNRPA
SNRPA1
SNRPB2
SNRPD1
SNRPD2
SNRPG
SRM
SRSF2
SRSF3
SRSF7
SSB
SSBP1
SYNCRIP
TARDBP
TCP1
TOMM70
TRA2B
TRIM28
TUFM
TXNL4A
UBE2L3
USP1
VBP1
XRCC6
YWHAE

PB

CKAP4
KLF2
KLF9
MXI1
PCGF5
RIN3
SEMA4B
UCKL1

NFKB

BCL2A1
CCL3
CCL4
DUSP2
EBI3
ID2
IL12B
LTA
NFKBIE
RGS1
TNF

NIK

ANXA4
C17orf49
CABLES1
CCDC28B
CFLAR
DOK5
EPB41L4B
HLA-DQA1
HLA-DRB1
HVCN1
IL21R
L1CAM
LMCD1
MGAT3
RASGRP3
SEMA4A
TRAF3IP3

Table S3. Gene sets (by biological process) overrepresented in MCL-PDLS compared to unstimulated (MCL-PB) controls

Biological process	# of enriched gene sets	NES (max)	FDR, q value (min)
**Proliferation**			
Cell cycle regulation	18	2,31	<0,001
DNA replication	31	2	<0,001
MYC regulated genes	1	1.64	0.008
KRAS pathway	1	1.51	0.024
**Immune pathways**			
Chemokines	5	2,11	<0,001
Allograft reaction	3	2,02	<0,001
Immune checkpoint control	1	1,97	0.002
Antigen presentation	9	1,91	0,013
Interleukins	3	1,84	0,007
Cytokines	2	1,75	0,035
Immune response	22	1,75	0.020
CD40-CD40L signalling	1	1,74	0,011
**Survival pathways**			
NFκB	8	1,94	0,001
TNF	3	1,75	0,037
**Cellular processes**			
Protein syntheis	10	3,01	0,001
RNA synthesis	4	1,87	0,006
Cell adhesion	2	1,82	0,002
Proteasome	2	1,71	0,011
**Metabolic pathways**			
Oxidative phosphorylation (OXPHOS)	13	1,96	0,002
Glucose metabolism	6	1,72	0,023
Fatty acid metabolism	4	1,72	0,023
Aminoacid metabolism	3	1,69	0,028
**DNA damage**			
DNA damage/repair	7	1,94	0,002
p53	1	1,65	0,04
**Others**			
Telomeres	2	1,77	0,016
Redox balance	4	1,73	0,017

GSEA was used to test for significant enrichment of defined gene signatures. NES indicates Normalized Enriched Score; FDR, False Discovery Rate. Threshold FDR<0.05 and NES>1.5. Custom genes set were experimentally derived and downloaded from http://lymphochip.nih.gov/signaturedb/index.html. C2CP, C3, C5 and H gene sets were obtained from the Molecular Signature Database (v2.5).

Table S4. Complete GSEA results of MCL-PDLS compared to unstimulated (MCL-PB) controls

GENE SET	SIZE	NES	FDR q-val
**Allograft reaction**			
KEGG_GRAFT_VERSUS_HOST_DISEASE	39	2.02	0.000
KEGG_ALLOGRAFT_REJECTION	37	1.94	0.000
HALLMARK_ALLOGRAFT_REJECTION	195	1.77	0.002
**Amminoacids Metabolism**			
KEGG_VALINE_LEUCINE_AND_ISOLEUCINE_DEGRADATION	44	1.64	0.023
KEGG_TRYPTOPHAN_METABOLISM	40	1.63	0.025
REACTOME_SELENOAMINO_ACID_METABOLISM	118	1,69	0.028
**Angiogenesis**			
HALLMARK_ANGIOGENESIS	33	1.61	0.010
**Antigen presentation**			
GOBP_ANTIGEN_PROCESSING_AND_PRESENTATION	107	1.91	0.013
REACTOME_ANTIGEN_PROCESSING_CROSS_PRESENTATION	106	1,86	0.006
GOBP_ANTIGEN_PROCESSING_AND_PRESENTATION_OF_ENDOGENOUS_ANTIGEN	26	1.84	0.032
GOBP_ANTIGEN_PROCESSING_AND_PRESENTATION_OF_EXOGENOUS_ANTIGEN	47	1.82	0.034
GOBP_PEPTIDE_ANTIGEN_ASSEMBLY_WITH_MHC_CLASS_II_PROTEIN_COMPLEX	16	1.83	0.036
GOBP_PEPTIDE_ANTIGEN_ASSEMBLY_WITH_MHC_PROTEIN_COMPLEX	20	1.82	0.034
MHC CLASS I	7	1.50	0.096
KEGG_ANTIGEN_PROCESSING_AND_PRESENTATION	73	1.76	0.006
REACTOME_CROSS_PRESENTATION_OF_SOLUBLE_EXOGENOUS_ANTIGENS_ENDOSOMES	50	1,8	0.012
**CD40-CD40L signalling**			
CD40 SIGNALLING DURING GC DEV	150	1.74	0.011
**Cell Adhesion**			
HALLMARK_APICAL_JUNCTION	197	1.55	0.018
KEGG_CELL_ADHESION_MOLECULES_CAMS	131	1.82	0.002
**Cell cycle regulation**			
HALLMARK_E2F_TARGETS	200	2.31	0.000
HALLMARK_G2M_CHECKPOINT	199	1.99	0.000
HALLMARK_MITOTIC_SPINDLE	199	1.59	0.013
KEGG_CELL_CYCLE	124	1.65	0.023
REACTOME_POLO_LIKE_KINASE_MEDIATED_EVENTS	16	1,73	0.021
REACTOME_APC_C_MEDIATED_DEGRADATION_OF_CELL_CYCLE_PROTEINS	88	1,94	0.002
REACTOME_CELL_CYCLE_CHECKPOINTS	289	1,92	0.003
REACTOME_G2_M_CHECKPOINTS	165	1,87	0.006
REACTOME_S_PHASE	162	1,84	0.007
REACTOME_G1_S_SPECIFIC_TRANSCRIPTION	29	1,82	0.011
REACTOME_MITOTIC_G1_PHASE_AND_G1_S_TRANSITION	149	1,81	0.012
REACTOME_CYCLIN_A_B1_B2_ASSOCIATED_EVENTS_DURING_G2_M_TRANSITION	25	1,72	0.023
REACTOME_TP53_REGULATES_TRANSCRIPTION_OF_GENES_INVOLVED_IN_G2_CELL_CYCLE_ARREST	18	1,71	0.023
REACTOME_MITOTIC_METAPHASE_AND_ANAPHASE	236	1,64	0.045
GOBP_REGULATION_OF_CHROMOSOME_SEPARATION	69	1.97	0.010
GOBP_MICROTUBULE_CYTOSKELETON_ORGANIZATION_INVOLVED_IN_MITOSIS	153	1.83	0.036
GOBP_MITOTIC_NUCLEAR_DIVISION	291	1.82	0.035
GOBP_MITOTIC_SPINDLE_ORGANIZATION	126	1.79	0.044
**Chemokines**			
REACTOME_CHEMOKINE_RECEPTORS_BIND_CHEMOKINES	53	1,86	0.006
GOMF_CCR_CHEMOKINE_RECEPTOR_BINDING	34	2.11	0.000
GOMF_CHEMOKINE_RECEPTOR_BINDING	54	2.08	0.000
GOMF_CHEMOKINE_ACTIVITY	40	2.07	0.000
GOMF_CHEMOATTRACTANT_ACTIVITY	29	1.73	0.047
**Cytokines**			
KEGG_CYTOKINE_CYTOKINE_RECEPTOR_INTERACTION	240	1.57	0.041
GOMF_CYTOKINE_ACTIVITY	205	1.75	0.035
**DNA damage/repair**			
KEGG_MISMATCH_REPAIR	23	1.77	0.005
KEGG_BASE_EXCISION_REPAIR	34	1.67	0.016
KEGG_NUCLEOTIDE_EXCISION_REPAIR	44	1.58	0.040
REACTOME_ACTIVATION_OF_ATR_IN_RESPONSE_TO_REPLICATION_STRESS	37	1,94	0.002
REACTOME_BASE_EXCISION_REPAIR	86	1,7	0.024
REACTOME_DNA_DOUBLE_STRAND_BREAK_REPAIR	164	1,63	0.046
REACTOME_MISMATCH_REPAIR	15	1,62	0.050
**DNA replication**			
KEGG_DNA_REPLICATION	36	2.00	0.000
REACTOME_DNA_STRAND_ELONGATION	32	2,02	0.001

REACTOME_SYNTHESIS_OF_DNA	120	2,01	0.000
REACTOME_CHROMOSOME_MAINTENANCE	135	1,97	0.002
REACTOME_DNA_REPLICATION	186	1,96	0.002
REACTOME_DEPOSITION_OF_NEW_CENPA_CONTAINING_NUCLEOSOMES_AT_THE_CENTROMERE	68	1,91	0.003
REACTOME_ACTIVATION_OF_THE_PRE_REPLICATIVE_COMPLEX	33	1,9	0.003
REACTOME_DNA_REPLICATION_PRE_INITIATION	143	1,88	0.005
REACTOME_MITOTIC_SPINDLE_CHECKPOINT	111	1,84	0.007
REACTOME_SEPARATION_OF_SISTER_CHROMATIDS	191	1,72	0.023
REACTOME_RESOLUTION_OF_SISTER_CHROMATID_COHESION	126	1,72	0.023
REACTOME_CONDENSATION_OF_PROPHASE_CHROMOSOMES	69	1,67	0.034
REACTOME_MITOTIC_PROMETAPHASE	203	1,67	0.035
GOBP_MONOCYTE_CHEMOTAXIS	62	2.08	0.000
GOBP_MITOTIC_SISTER_CHROMATID_SEGREGATION	171	1.98	0.012
GOBP_METAPHASE_ANAPHASE_TRANSITION_OF_CELL_CYCLE	63	1.96	0.009
GOBP_REGULATION_OF_CHROMOSOME_SEGREGATION	86	1.96	0.008
GOBP_SISTER_CHROMATID_SEGREGATION	203	1.96	0.007
GOBP_CENTROMERE_COMPLEX_ASSEMBLY	31	1.94	0.008
GOBP_KINETOCHORE_ORGANIZATION	23	1.93	0.010
GOBP_REGULATION_OF_MITOTIC_SISTER_CHROMATID_SEGREGATION	45	1.92	0.011
GOBP_CHROMOSOME_SEPARATION	94	1.91	0.013
GOBP_CHROMOSOME_SEGREGATION	343	1.89	0.017
GOBP_NUCLEAR_CHROMOSOME_SEGREGATION	284	1.89	0.016
GOBP_NEGATIVE_REGULATION_OF_METAPHASE_ANAPHASE_TRANSITION_OF_CELL_CYCLE	41	1.86	0.026
GOBP_DNA_REPLICATION_INITIATION	38	1.81	0.037
GOCC_NUCLEAR_REPLICATION_FORK	28	1.85	0.007
GOCC_REPLICATION_FORK	60	1.84	0.007
GOCC_CONDENSED_CHROMOSOME_CENTROMERIC_REGION	156	1.83	0.006
GOCC_CHROMOSOME_CENTROMERIC_REGION	226	1.76	0.014
GOCC_REPLISOME	15	1.69	0.023
**Fatty Acid metabolism**			
KEGG_BIOSYNTHESIS_OF_UNSATURATED_FATTY_ACIDS	22	1.60	0.037
KEGG_FATTY_ACID_METABOLISM	42	1.59	0.042
REACTOME_FATTY_ACID_METABOLISM	177	1,72	0.023
REACTOME_MITOCHONDRIAL_FATTY_ACID_BETA_OXIDATION	37	1,64	0.044
**Glucose metabolism**			
HALLMARK_GLYCOLYSIS	199	1.57	0.015
KEGG_GLYCOLYSIS_GLUCONEOGENESIS	61	1.56	0.046
KEGG_ONE_CARBON_POOL_BY_FOLATE	17	1.55	0.050
KEGG_PENTOSE_PHOSPHATE_PATHWAY	26	1.54	0.050
REACTOME_GLUCONEOGENESIS	33	1,72	0.023
KEGG_PYRUVATE_METABOLISM	40	1.58	0.041
**Immune checkpoint control**			
REACTOME_PD_1_SIGNALING	23	1,97	0.002
**Immune response**			
REACTOME_IMMUNOREGULATORY_INTERACTIONS_BETWEEN_A_LYMPHOID_AND_A_NON_LYMPHC	184	1,75	0.020
REACTOME_INTERFERON_GAMMA_SIGNALING	92	1,74	0.021
GOBP_ANTIGEN_PROCESSING_AND_PRESENTATION	107	1.91	0.013
GOBP_ANTIGEN_PROCESSING_AND_PRESENTATION_OF_ENDOGENOUS_ANTIGEN	26	1.84	0.032
GOBP_LYMPHOCYTE_MEDIATED_IMMUNITY	351	1.83	0.034
GOBP_PEPTIDE_ANTIGEN_ASSEMBLY_WITH_MHC_CLASS_II_PROTEIN_COMPLEX	16	1.83	0.036
GOBP_PEPTIDE_ANTIGEN_ASSEMBLY_WITH_MHC_PROTEIN_COMPLEX	20	1.82	0.034
EPTORS_BUILT_FROM_IMMUNOGLOBULIN_SUPERFAMILY_DOMAINS	357	1.82	0.034
GOBP_ANTIGEN_PROCESSING_AND_PRESENTATION_OF_EXOGENOUS_ANTIGEN	47	1.82	0.034
GOBP_MACROPHAGE_MIGRATION	53	1.80	0.042
GOBP_CELLULAR_RESPONSE_TO_INTERFERON_GAMMA	113	1.80	0.043
GOBP_T_CELL_MEDIATED_IMMUNITY	111	1.79	0.046
GOBP_B_CELL_MEDIATED_IMMUNITY	208	1.79	0.049
GOCC_MHC_PROTEIN_COMPLEX	26	1.91	0.003
GOCC_IMMUNOGLOBULIN_COMPLEX_CIRCULATING	67	1.85	0.007
GOCC_T_CELL_RECEPTOR_COMPLEX	39	1.84	0.006
GOCC_MHC_CLASS_II_PROTEIN_COMPLEX	17	1.78	0.011
GOCC_IMMUNOGLOBULIN_COMPLEX	146	1.76	0.014
GOMF_ANTIGEN_BINDING	156	1.87	0.012
GOMF_MHC_PROTEIN_COMPLEX_BINDING	36	1.83	0.022
GOMF_MHC_CLASS_II_PROTEIN_COMPLEX_BINDING	27	1.80	0.027
GOMF_IMMUNOGLOBULIN_RECEPTOR_BINDING	70	1.78	0.031

**Interleukins**			
IL17PATHWAY	14	1.64	0.031
REACTOME_INTERLEUKIN_10_SIGNALING	46	1,84	0.007
REACTOME_INTERLEUKIN_4_AND_INTERLEUKIN_13_SIGNALING	110	1,63	0.048
**KRAS pathway**			
HALLMARK_KRAS_SIGNALING_UP	195	1.51	0.024
**MYC regulates genes**			
HALLMARK_MYC_TARGETS_V1	200	1.64	0.008
**NFkB**			
NFKB TARGET GENES	23	1.98	0.000
NFKB _DLBCL NATURE 2009_COMPAGNO	109	1.94	0.001
NFKB_ALL_OCILY3_LY10	57	1.87	0.003
NIK SIGNATURE	26	1.84	0.003
NFKB PATHWAY MODULATED BY IBRUTINIB	39	1.83	0.004
NFKB_BOTHOCILY3ANDLY10	33	1.76	0.010
NFKB SIGNATURE	18	1.74	0.011
REACTOME_DECTIN_1_MEDIATED_NONCANONICAL_NF_KB_SIGNALING	62	1,81	0.011
**Oxidative phosphorylation**			
HALLMARK_OXIDATIVE_PHOSPHORYLATION	200	1.86	0.001
KEGG_OXIDATIVE_PHOSPHORYLATION	129	1.71	0.011
REACTOME_MITOCHONDRIAL_TRANSLATION	94	1,96	0.002
REACTOME_RESPIRATORY_ELECTRON_TRANSPORT	103	1,73	0.022
GOBP_MITOCHONDRIAL_RESPIRATORY_CHAIN_COMPLEX_ASSEMBLY	94	1.86	0.025
GOBP_OXIDATIVE_PHOSPHORYLATION	136	1.84	0.034
GOBP_ELECTRON_TRANSPORT_CHAIN	165	1.78	0.049
GOCC_MITOCHONDRIAL_PROTEIN_CONTAINING_COMPLEX	279	1.93	0.002
GOCC_INNER_MITOCHONDRIAL_MEMBRANE_PROTEIN_COMPLEX	153	1.81	0.008
GOCC_PROTON_TRANSPORTING_ATP_SYNTHASE_COMPLEX	21	1.73	0.017
GOCC_RESPIRASOME	100	1.71	0.018
GOCC_NADH_DEHYDROGENASE_COMPLEX	49	1.62	0.044
GOMF_ELECTRON_TRANSFER_ACTIVITY	124	1.77	0.030
**p53**			
REACTOME_STABILIZATION_OF_P53	57	1,65	0.040
**Proliferation**			
MCL_PROLIFERATION_SIGNATURE	14	1.90	0.002
**Proteasome**			
KEGG_PROTEASOME	44	1.71	0.011
GOCC_PROTEASOME_CORE_COMPLEX	20	1.70	0.022
**Protein syntheis**			
KEGG_RIBOSOME	88	1.82	0.002
GOCC_RIBOSOMAL_SUBUNIT	177	2.01	0.001
GOCC_ORGANELLAR_RIBOSOME	81	1.99	0.000
GOCC_LARGE_RIBOSOMAL_SUBUNIT	111	1.96	0.001
GOCC_MITOCHONDRIAL_LARGE_RIBOSOMAL_SUBUNIT	54	1.90	0.003
GOCC_RIBOSOME	208	1.83	0.007
GOCC_SMALL_RIBOSOMAL_SUBUNIT	69	1.74	0.015
GOCC_CYTOSOLIC_RIBOSOME	101	1.71	0.019
GOCC_CYTOSOLIC_LARGE_RIBOSOMAL_SUBUNIT	57	1.69	0.023
REACTOME_EUKARYOTIC_TRANSLATION_INITIATION	120	1,65	0.040
**Redox balance**			
KEGG_PEROXISOME	78	1.59	0.040
REACTOME_DETOXIFICATION_OF_REACTIVE_OXYGEN_SPECIES	36	1,64	0.042
GOCC_OXIDOREDUCTASE_COMPLEX	120	1.73	0.017
AS_ACCEPTOR	57	1.73	0.046
**RNA synthesis**			
REACTOME_LAGGING_STRAND_SYNTHESIS	20	1,87	0.006
REACTOME_EUKARYOTIC_TRANSLATION_ELONGATION	94	1,86	0.006
REACTOME_TELOMERE_C_STRAND_LAGGING_STRAND_SYNTHESIS	34	1,82	0.010
REACTOME_TRANSLATION	293	1,8	0.013
**Telomeres**			
REACTOME_EXTENSION_OF_TELOMERES	51	1,77	0.016
REACTOME_TELOMERE_MAINTENANCE	108	1,75	0.018
**TNF**			
REACTOME_TNFS_BIND_THEIR_PHYSIOLOGICAL_RECEPTORS	29	1,69	0.027
REACTOME_TNFR2_NON_CANONICAL_NF_KB_PATHWAY	101	1,67	0.033
GOMF_TUMOR_NECROSIS_FACTOR_RECEPTOR_BINDING	31	1.75	0.037

Table S5. Complete GSEA results of MCL-PDLS compared to MCL-2D co-cultures

GENE SET	SIZE	NES	FDR q-val	CLASS
REACTOME_CROSS_PRESENTATION_OF_SOLUBLE_EXOGENOUS_ANTIGENS_ENDOSOMES	50	1.88	0.048	Ag presentation
HALLMARK_ANGIOGENESIS	33	1.72	0.042	Angiogenesis
REACTOME_ADHERENS_JUNCTIONS_INTERACTIONS	33	1.91	0.028	Cell adhesion
REACTOME_SCF_SKP2_MEDIATED_DEGRADATION_OF_P27_P21	60	1.87	0.044	Cell cycle
REACTOME_CDT1_ASSOCIATION_WITH_THE_CDC6_ORC_ORIGIN_COMPLEX	59	1.90	0.032	DNA replication
REACTOME_EXTRACELLULAR_MATRIX_ORGANIZATION	295	1.98	0.003	Extracellular matrix
REACTOME_ACTIVATION_OF_MATRIX_METALLOPROTEINASES	31	1.89	0.038	Extracellular matrix
GOCC_COLLAGEN_CONTAINING_EXTRACELLULAR_MATRIX	413	2.03	<0.001	Extracellular matrix
GOCC_COLLAGEN_TRIMER	84	1.94	0.009	Extracellular matrix
GOMF_EXTRACELLULAR_MATRIX_STRUCTURAL_CONSTITUENT	165	2.08	<0.001	Extracellular matrix
GOMF_COLLAGEN_BINDING	65	2.01	0.002	Extracellular matrix
REACTOME_DECTIN_2_FAMILY	26	1.89	0.039	Immune response
HALLMARK_EPITHELIAL_MESENCHYMAL_TRANSITION	198	1.72	0.034	Metastasis
BIOCARTA_RACC_PATHWAY	15	1.90	0.048	Ion channel activity
GOMF_EXTRACELLULAR_LIGAND_GATED_ION_CHANNEL_ACTIVITY	73	1.93	0.023	Ion channel activity
GOMF_LIGAND_GATED_ION_CHANNEL_ACTIVITY	144	1.90	0.044	Ion channel activity
GOCC_ION_CHANNEL_COMPLEX	286	1.93	0.009	Ion channel activity
HALLMARK_KRAS_SIGNALING_UP	195	1.84	0.015	KRAS
HALLMARK_MYC_TARGETS_V1	200	1.83	0.010	MYC
REACTOME_NEGATIVE_REGULATION_OF_NOTCH4_SIGNALING	54	1.88	0.042	Notch pathway
E47_02	249	1.72	0.014	Stemness
KEGG_PROTEASOME	44	1.97	0.004	Proteasome
REACTOME_DISEASES_ASSOCIATED_WITH_O_GLYCOSYLATION_OF_PROTEINS	68	1.98	0.003	Protein glycosylation
REACTOME_O_GLYCOSYLATION_OF_TSR_DOMAIN_CONTAINING_PROTEINS	39	1.94	0.012	Protein glycosylation
REACTOME_DEFECTIVE_GALNT3_CAUSES_HFTC	16	1.92	0.026	Protein glycosylation
REACTOME_TERMINATION_OF_O_GLYCAN_BIOSYNTHESIS	23	1.91	0.028	Protein glycosylation
REACTOME_DEFECTIVE_C1GALT1C1_CAUSES_TNPS	17	1.87	0.046	Protein glycosylation

GSEA was used to test for significant enrichment of defined gene signatures. NES indicates Normalized Enriched Score; FDR, False Discovery Rate. Threshold FDR<0.05 and NES>1.5. Custom genes set were experimentally derived and downloaded from http://lymphochip.nih.gov/signaturedb/index.html. C2CP, C3, C5 and H gene sets were obtained from the Molecular Signature Database (v2.5).

Table S6. Leading edge genes of specific signatures from Figure 3E

Myc

AIMP2
AP3S1
APEX1
C1QBP
CBX3
CCT2
CCT3
CCT4
CCT5
CCT7
CDC20
CDC45
CDK4
CLNS1A
CNBP
COPS5
COX5A
CTPS1
DEK
EEF1B2
EIF1AX
EIF2S1
EIF2S2
EIF3B
EIF4A1
EIF4E
ERH
EXOSC7
FBL
GLO1
GNL3
GOT2
GSPT1
H2AZ1
HDAC2
HDGF
HNRNPA1
HNRNPA2B1
HNRNPA3
HNRNPC
HNRNPD
HNRNPR
HNRNPU
HPRT1
HSP90AB1
HSPD1
HSPE1
IARS1
IFRD1
ILF2
KARS1
KPNA2
LDHA
LSM7
MAD2L1
MCM2
MCM4
MCM6
MCM7
MRPL23
MRPL9
MRPS18B
NDUFAB1
NHP2
NME1
NOLC1
NOP16
NPM1
ODC1
PCNA
POLD2
POLE3
PPIA
PPM1G
PRDX4
PRPF31
PSMA1
PSMA2
PSMA4
PSMA6
PSMA7
PSMB2
PSMB3
PSMC4
PSMC6
PSMD1
PSMD14
PSMD3
PSMD7
PTGES3
PWP1
RAN
RANBP1
RFC4
RPL18
RPL34
RPL6
RPS2
RPS5
RRM1
RRP9
RUVBL2
SERBP1
SET
SF3B3
SLC25A3
SNRPA
SNRPA1
SNRPB2
SNRPD1
SNRPD2
SNRPG
SRM
SRSF2
SRSF3
SRSF7
SSB
SSBP1
SYNCRIP
TARDBP
TCP1
TOMM70
TRA2B
TRIM28
TUFM
TXNL4A
UBE2L3
USP1
VBP1
XRCC6
YWHAE

Collagen

C1QB
C1QC
CCDC80
COL12A1
COL6A3
COL7A1
CRELD1
CTSL
EGFLAM
FRAS1
HSP90B1
ITIH4
LGALS9
LTBP3
MMP9
PTPRZ1
S100A4
SEMA3B
THBS1
TNC
USH2A
VWF
WNT5B
ZP3

ECM org

COL12A1
COL22A1
COL6A3
COL7A1
CTSL
LRP4
MMP16
MMP19
MMP9
NRXN1
SPP1
THBS1
TNC
VWF

E47_02

GABRE
COL22A1
MMP16
DOC2B
BTBD3
CASKIN2
FGF13
ELP5
ERBB3
SNAP25
TPI1
